# Maximizing statistical power to detect differentially abundant cell states with scPOST

**DOI:** 10.1016/j.crmeth.2021.100120

**Published:** 2021-11-22

**Authors:** Nghia Millard, Ilya Korsunsky, Kathryn Weinand, Chamith Y. Fonseka, Aparna Nathan, Joyce B. Kang, Soumya Raychaudhuri

**Affiliations:** 1 Center for Data Sciences, Brigham and Women’s Hospital, Boston, MA 02115, USA; 2 Division of Genetics, Department of Medicine, Brigham and Women’s Hospital and Harvard Medical School, Boston, MA 02115, USA; 3 Division of Rheumatology, Inflammation, and Immunity, Department of Medicine, Brigham and Women’s Hospital and Harvard Medical School, Boston, MA 02115, USA; 4 Department of Biomedical Informatics, Harvard Medical School, Boston, MA 02115, USA; 5 Program in Medical and Population Genetics, Broad Institute of MIT and Harvard, Cambridge, MA 02142, USA; 6 Versus Arthritis Centre for Genetics and Genomics, Centre for Musculoskeletal Research, Manchester Academic Health Science Centre, The University of Manchester, Manchester 46962, UK; 7 Lead contact

## Abstract

To estimate a study design’s power to detect differential abundance, we require a framework that simulates many multi-sample single-cell datasets. However, current simulation methods are challenging for large-scale power analyses because they are computationally resource intensive and do not support easy simulation of multi-sample datasets. Current methods also lack modeling of important inter-sample variation, such as the variation in the frequency of cell states between samples that is observed in single-cell data. Thus, we developed single-cell POwer Simulation Tool (scPOST) to address these limitations and help investigators quickly simulate multi-sample single-cell datasets. Users may explore a range of effect sizes and study design choices (such as increasing the number of samples or cells per sample) to determine their effect on power, and thus choose the optimal study design for their planned experiments.

## In brief

Millard et al. present single-cell POwer Simulation Tool (scPOST), a tool that allows investigators to simulate single-cell datasets in order to perform power analyses for study design optimization, as well as compare and assess algorithm performance.

## Graphical Abstract

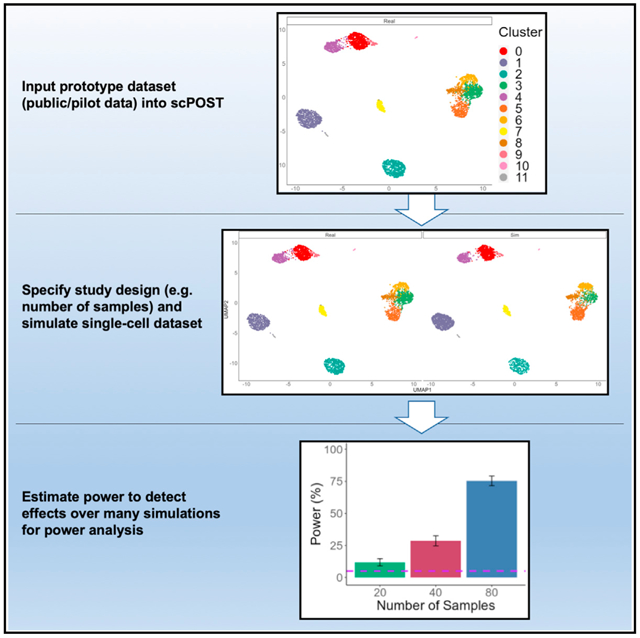

## INTRODUCTION

Single-cell technologies are revolutionizing biological studies. For example, single-cell RNA sequencing (scRNA-seq) can measure the transcriptome of individual cells (Tang et al., 2009, Tanay and Regev, 2017) to describe previously unobserved cellular heterogeneity (Jaitin et al., 2014; Shalek et al., 2014). Technological advances allowing for simultaneous measurement of an increasing diversity of modalities (Cusanovich et al., 2015; Chen et al., 2015; Stoeckius et al., 2017; Rodriques et al., 2019) from increasing numbers of cells (Svensson et al., 2018) are enabling disease-focused single-cell studies to identify cell states, often defined as clusters, whose frequency correlates with disease in blood or tissue (Zhang et al., 2019a; Smillie et al., 2019; Kotliarov et al., 2020; Schafflick et al., 2020; Nathan et al., 2021). Detecting clusters that are differentially abundant (DA) between sample conditions (such as diseased versus healthy) aids researchers in focusing on potential disease mechanisms because DA clusters are often associated with biologically meaningful phenotypes; expanded populations in disease states are often associated with pathogenic mechanisms, which can be further defined by differential gene expression programs (Soneson and Robinson, 2018). For example, a recent study identified and characterized a CD14^+^ monocyte cluster expanded in individuals with sepsis versus healthy controls (Reyes et al., 2020), which helped focus downstream experiments on that cluster. Furthermore, DA clusters have also been associated with genetic variants (Orrù et al., 2013), highlighting the potential to associate genetic variants with mechanisms that affect cluster abundance. In order to efficiently detect DA clusters, it is essential to conduct large, well-powered case-control comparisons (Hollenbach et al., 2014).

In this study, we consider a wide range of factors that potentially affect power: variation in cluster frequencies across samples, gene expression variation, batch structure, numbers of cells and samples, and sequencing depth. Due to variation across studies, these factors must be simulated in the study’s context to accurately estimate power.

Current single-cell simulation strategies directly simulate individual genes and focus on estimating power to detect differential gene expression (Zappia et al., 2017; Vieth et al., 2017; Zhang et al., 2019b, Li and Li, 2019) or associations with genetic variants (Schmid et al., 2020; Mandric et al., 2020). These strategies are typically used to simulate small datasets ranging from 400 to 10,000 cells in a single sample with few replicates because sampling individual genes is slow. Furthermore, no strategies model the variation in cluster frequencies across samples that is observed in single-cell data, which is critical for estimating power to detect DA clusters.

Here, we present single-cell POwer Simulation Tool (scPOST), a framework that enables the fast simulation of multi-sample datasets with >25,000 cells spanning hundreds of samples, which is not feasible with current methods. scPOST models inter-sample cluster frequency variation and generates datasets based on (1) batch and sample parameters learned from real input prototype data (public or pilot), and (2) user-specified scaling factors. scPOST can model large studies based on minimal input data and allows users to modulate the parameters of the simulations, which ultimately informs optimal study design. We first show that scPOST can simulate new datasets that mimic the structure of the original data and then apply scPOST to three diverse single-cell datasets, where we modify study design parameters with the overall goal of guiding the design of larger studies that maximize power.

## RESULTS

### Summary of statistical approach

**Figure 1.**
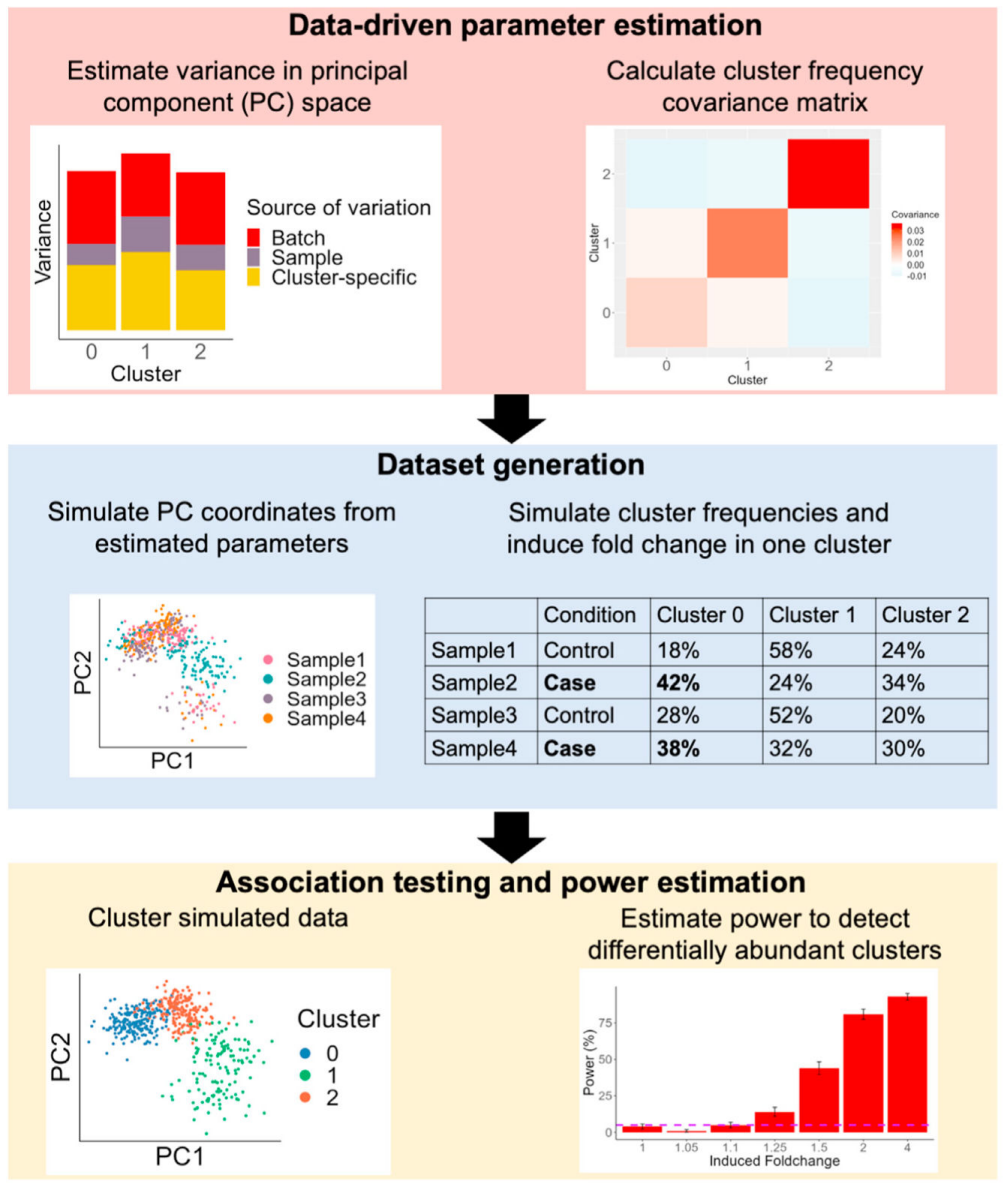
scPOST simulates single-cell datasets to estimate power to detect differential abundance in a cluster between conditions scPOST comprises three steps: data-driven parameter estimation, dataset simulation, and association testing. scPOST models gene expression variation (left) and cluster frequency variation (right).

scPOST comprises three steps: (1) parameter estimation from a prototype dataset, (2) simulation of datasets based on estimated parameters, and (3) power calculations from DA testing on the simulated data (Figure 1).

scPOST relies on user-provided prototype data to estimate key parameters used for simulation. Prototype data should reflect the planned experimental setting, including the single-cell platform being used, assayed cell states, and sample type. We recommend that prototype data include a minimum of six samples with at least 500 cells per sample so that it properly captures the inter-sample cell state and gene expression covariation of the expected data. Users can modify key study design factors, including number of cell states, number of samples, number of cells per sample, multiplexing structure, and magnitude of simulated noise.

**Step 1:** We model gene expression in low-dimensional principal components (PCs) rather than high-dimensional measurements of individual genes because PCs capture gene covariation and are often the input for downstream analyses like clustering (Luecken and Theis, 2019). Input data for scPOST typically consists of PCs and cell state classifications (any arbitrary grouping of cells, represented here as clusters derived from Louvain clustering (Blondel et al., 2008; Levine et al., 2015). We model PC variation in a cluster-specific manner since variance (such as batch-associated variation) can be different for each cluster; these differential variances are often modeled and corrected for in batch-correction algorithms (Haghverdi et al., 2018; Korsunsky et al., 2019).

**Figure 2.**
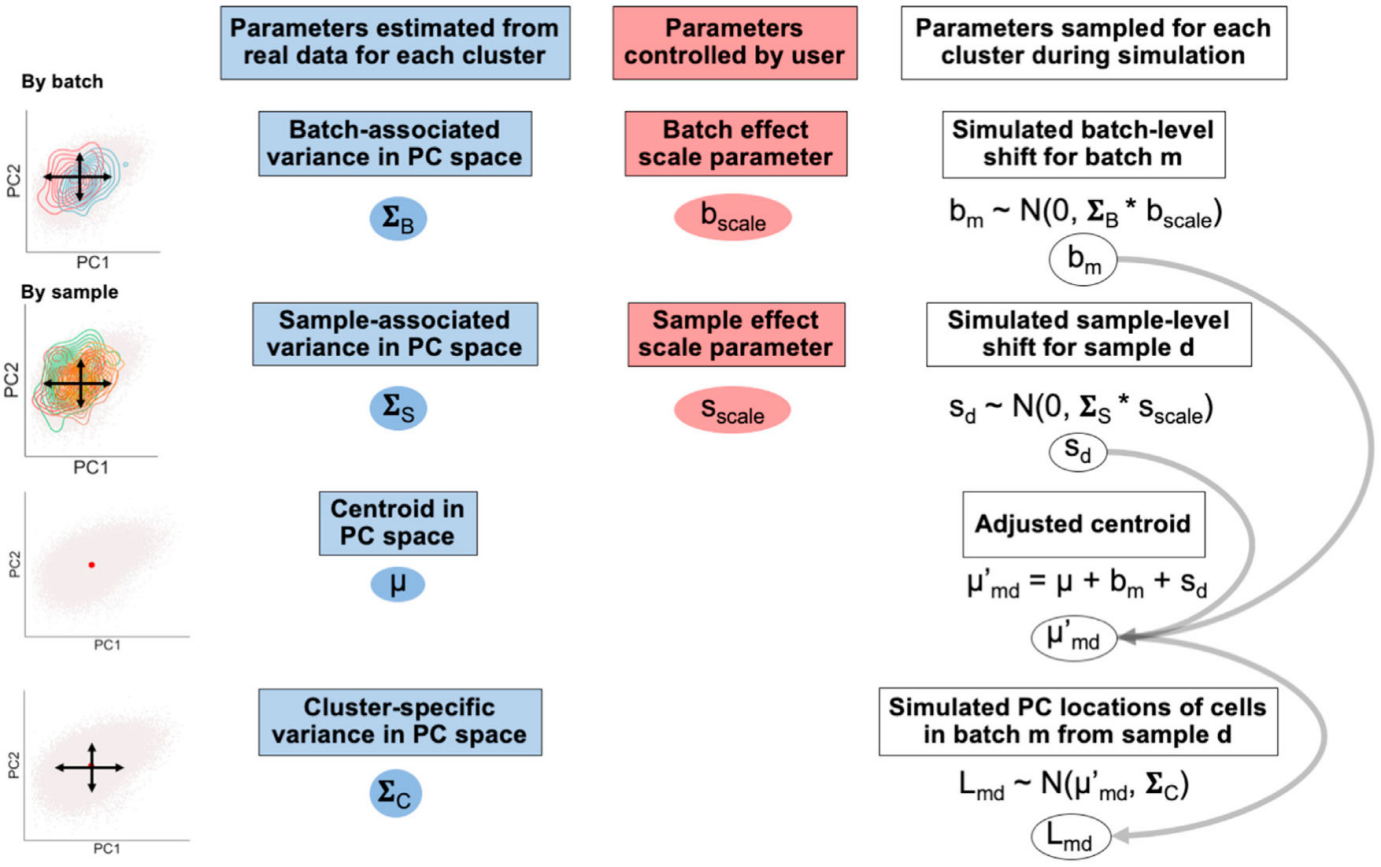
scPOST simulates gene expression by sampling PC coordinates for each cell For each cluster, we simulate batch- and sample-specific shifts with cluster-specific parameters estimated from real data (blue) and user-controlled scaling factors (red). We sum these shifts with the cluster-specific centroid to obtain an adjusted centroid, which we use with the residual cluster-specific variation to sample PC coordinates.

For each cluster, we estimate the variance in PC space captured by batch (Σ_B_) and sample identity (Σ_S_) with linear mixed effects models that have batch and sample identity fitted as independent random effects (Figure 2, STAR Methods). From the same models, we retrieve each cluster’s unconditioned centroid in PC space (μ), representing that cluster’s mean value along each PC. We also obtain the residual variance (Σ_C_), representing cluster-specific variance not attributed to batch or sample identity. From the input dataset’s cluster frequency distributions, we estimate each cluster’s log mean frequency across samples (μ_cf_) and log covariance with other clusters (Σ_cf_) (Figure S1A). By default, we include cluster frequency covariances because certain clusters may covary (e.g., the frequencies of T cells and B cells may covary).

**Step 2:** During simulation, we simulate random linear shifts in a cluster-specific manner for each batch *m* (*b*_*m*_) and each sample *d* (*s_d_*) from the estimated parameters (Σ_B_) and (Σ_S_), respectively; these shifts are multiplied by user-controlled scaling factors (b_scale_ and s_scale_, respectively) to modulate the magnitude of these effects. These shifts produce an adjusted centroid (μ′_md_) for each batch-sample pair. Finally, we sample a PC coordinate for each cell from a multivariate normal distribution centered at the batch-sample adjusted centroid (μ′_md_) with the estimated cluster-specific residual covariance (Σ_C_) (Figure 2). We simulate each sample’s cluster frequency distribution by sampling from a multivariate normal distribution parameterized by (μ_cf_) and (Σ_cf_), respectively (STAR Methods). To simulate DA in cluster frequencies, the user may choose a cluster to induce a fold change (effect size) difference between conditions (e.g., a 2-fold expansion in cases versus controls); otherwise, a cluster will be randomly assigned. We encourage users to test a range of fold changes to obtain a realistic idea of effect sizes they are likely to detect.

**Step 3:** To estimate power, we independently cluster the cells in each simulated dataset and test for DA with Mixed effects Association testing for Single Cells (MASC; Fonseka et al., 2018). In brief, MASC fits logistic mixed effects models on single-cell data and then performs a likelihood ratio test to determine whether a full model containing case-control status explains a cell’s cluster membership significantly better than a null model without case-control status. For each simulation, we ran MASC on each cluster and checked if any cluster p value passed a Bonferroni-corrected threshold of p < 0.05/*k*, where *k* is the number of simulated clusters. In a simulation, we considered detection successful when at least one cluster had significant DA between conditions (STAR Methods). We present power as the percentage of successful simulations, e.g., 50% power indicates successful detection in 250 of 500 simulations.

### scPOST estimates parameters from three input prototype datasets

**Figure 3.**
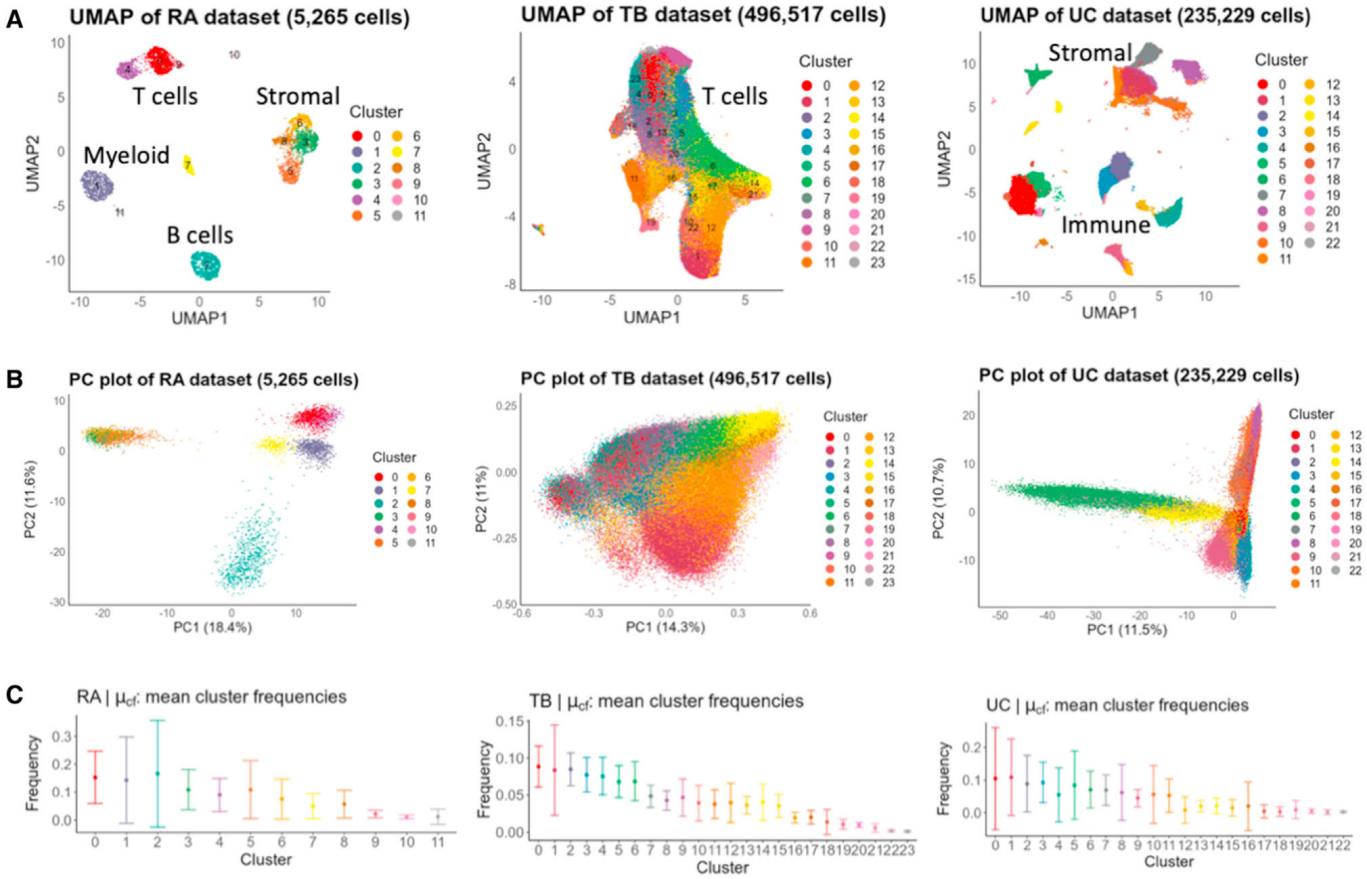
The RA, TB, and UC datasets feature a diverse set of cell types, PC structures, and cluster frequencies (A) UMAP visualizations of the RA, TB, and UC datasets colored by cluster. (B) PC plots of each datasets highlight differences in PC structure, e.g., differing scale of PC values. (C) Frequency distributions for each cluster. Dots represent the observed mean frequency of that cluster across all samples in their respective dataset. In all panels, error bars represent 1 SD from the mean in each direction to showcase the spread of frequencies across samples.

To demonstrate the utility of scPOST, we applied our framework to three independent scRNA-seq datasets: a rheumatoid arthritis (RA) dataset containing 5,265 immune and stromal cells from 21 synovial tissue samples (Zhang et al., 2019a), a tuberculosis (TB) dataset containing 496,517 peripheral blood memory T cells from 259 peripheral blood mononuclear samples (Nathan et al., 2021), and an ulcerative colitis (UC) dataset containing 235,229 immune and stromal cells from 30 intestinal biopsies (Smillie et al., 2019). These data vary in scRNA-seq technology utilized, tissues assayed, number of cells, number of samples, and number of clusters we derived from Louvain clustering (Figure 3A, Table S1).

scPOST parameter estimation highlights the diversity of structure in single-cell datasets. For example, the RA and UC datasets featured higher variance in PC space (Figures 3B and S1B), which likely reflects the presence of multiple distinct broad cell types. Furthermore, the cluster frequency variation between samples in the RA and UC datasets tended to be higher in more clusters compared with the TB dataset (Figure 3C), perhaps since they are derived from more heterogeneous dissociated tissue compared with the less destructive collection of blood.

### scPOST simulates datasets that mimic gene expression structure found in the input prototype data

**Figure 4.**
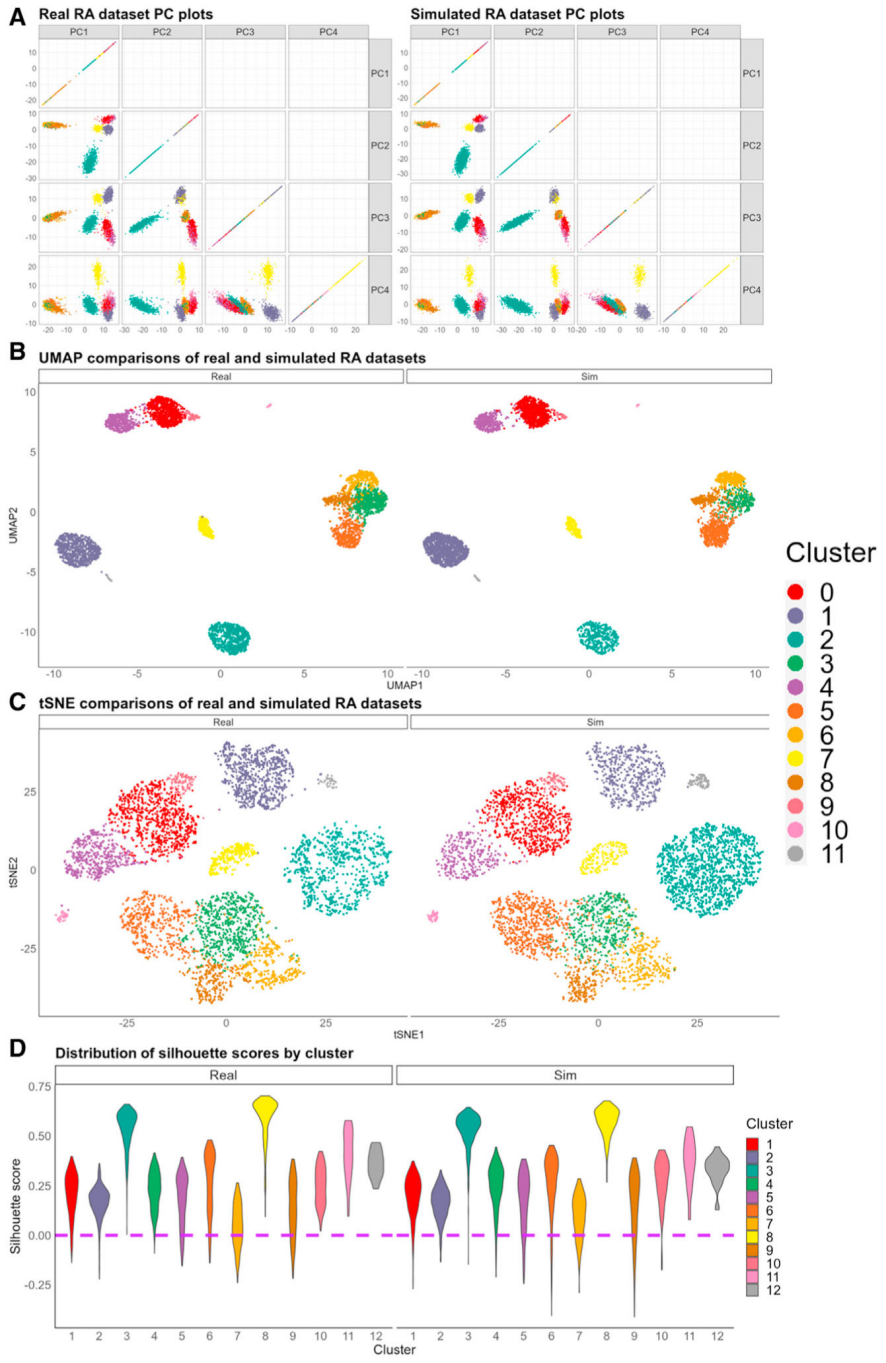
scPOST can simulate realistic datasets with PC structure similar to the input RA dataset (A) PC plots comparing the real RA input dataset with a realistic simulated dataset generated by scPOST. (B and C) UMAP or tSNE visualizations of the real RA dataset and the simulated dataset, which were embedded into the same respective UMAP/tSNE space based off PCs. (D) Violin plots comparing the distribution of silhouette scores between the real RA dataset and the simulated dataset.

PCs are commonly used to capture the structure of scRNA-seq data. To evaluate the gene expression structure of simulated datasets generated by scPOST, we first used the RA dataset as input. We simulated a realistic, similarly sized dataset (5,250 cells, STAR Methods) and compared PC plots between the real RA data and the simulated RA data (Figure 4A). Unsurprisingly, the PC plots between the two datasets are extremely similar, which suggests that the simulated PC structure mimics the real dataset’s structure. We then performed two independent dimensionality reductions using either uniform manifold approximation and projection (UMAP; McInnes et al., 2018) or t-distributed stochastic neighbor embedding (tSNE; Van Der Maaten and Hinton, 2008) to independently embed the cells from the two datasets into 2-dimensional space. Comparing the UMAP embeddings or the tSNE embeddings between the real and the simulated datasets again shows that the structure between the two is qualitatively similar (Figures 4B and 4C). Finally, we calculated silhouette scores, a measure of intra- versus inter-cluster variability per cell, for each cell in both datasets (Figure 4D). Notably, the simulated data have similar silhouette distributions, even in clusters with lower silhouette scores (e.g., cluster 7). We then used scPOST to simulate realistic, similarly sized data from the TB and UC datasets (496,503 and 235,200 cells, respectively). Again, comparing PC, UMAP, and tSNE plots of the simulated datasets with their respective input data showed similarity in structure (Figures S2A and S2B). We concluded that our strategy can generate realistic datasets that are similar in gene expression structure to the input data.

### scPOST simulates multi-sample datasets that feature cluster frequency variation

In addition to simulating gene expression structure, scPOST simulates multi-sample datasets whose samples have varying cluster frequency distributions. When simulating realistic datasets, scPOST can generate cluster frequency distributions similar to the real data (Figure S2D). scPOST allows users to simulate cluster-specific expansions between conditions (e.g., a 2-fold expansion in a cluster between cases and controls). Due to cluster frequency variation between samples in a simulation, the observed fold change in a cluster may vary from the intended fold change. From simulations derived from the RA data, we show that with realistic cluster frequency variation (cf_scale_ = 1), the spread of observed fold changes across simulations can be highly variable, but reducing the cf_scale_ parameter leads to less variability (Figure S2E). When cf_scale_ is equal to 0, there is no cluster frequency variation between samples, meaning that all samples within a condition have the same cluster frequency distribution and the observed fold change between conditions matches the induced fold change.

### scPOST estimates power benefits from expanding a RA study

**Figure 5.**
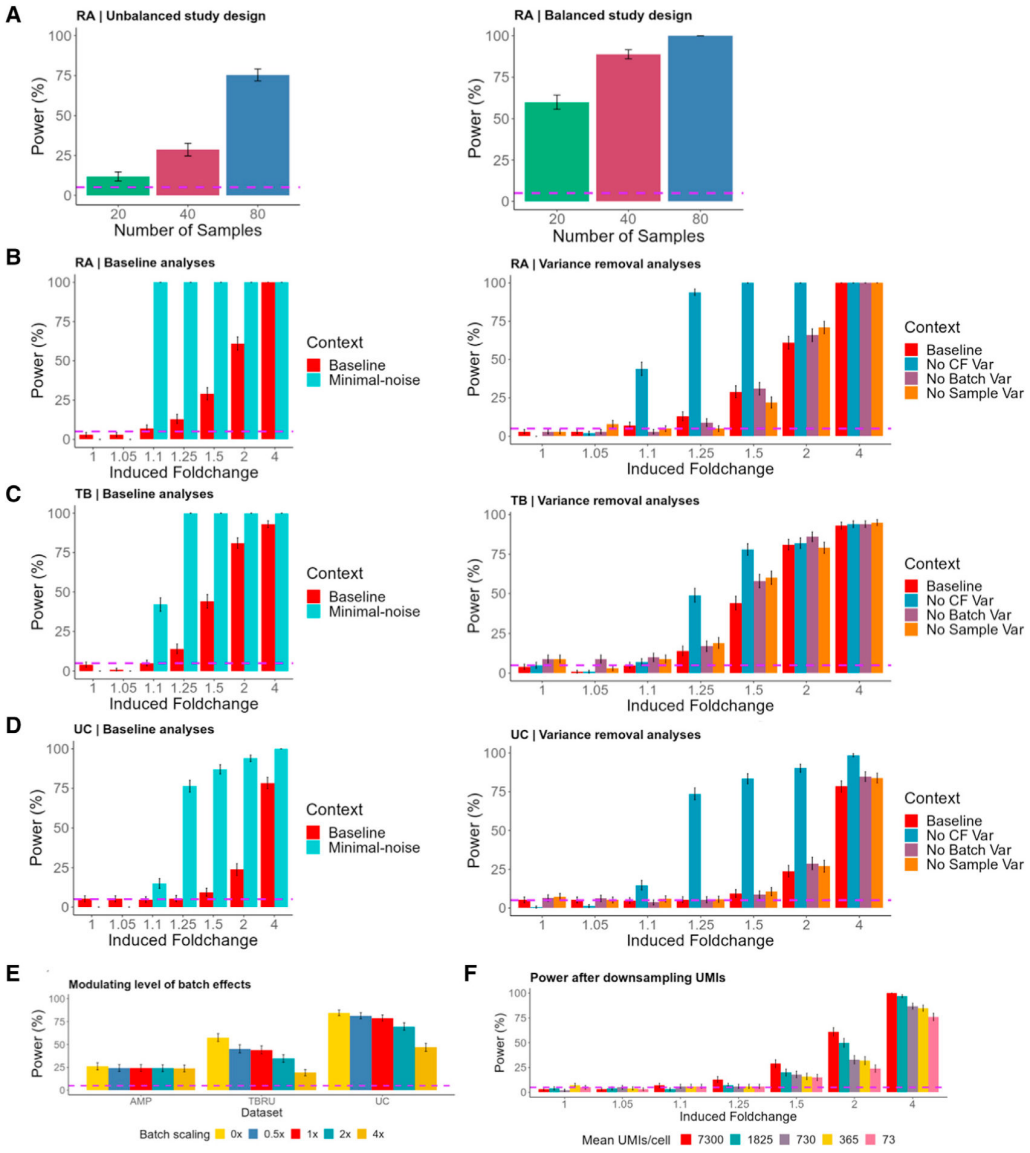
scPOST estimates power from multiple study designs Each bar represents n = 500 simulations. Error bars represent 95% binomial proportion confidence intervals; dotted horizontal purple lines are set at 5%. (A) Results from increasing the size of a study. (B–D) Left, Baseline/minimal-noise analyses for the RA, TB, and UC datasets. Baseline context features realistic levels of cluster frequency (CF) variation and gene expression variation (b_scale_ = 1, s_scale_ = 1, cf_scale_ = 1). Minimal-noise removes these sources of variation (e.g., b_scale_ = 0). Right, analyses in which only one source of variation (CF, batch, or sample) is removed. (E) Batch scaling simulations: 0× indicates no batch variation, whereas 4× indicates 4 times the level of estimated realistic batch variation. Induced fold changes of 1.5 in RA and TB settings and fold change of 4 in UC. (F) Power calculations derived from downsampling the RA UMI data (red = baseline).

To highlight the value of scPOST in evaluating different study designs, we used scPOST to determine how best to expand the previously described RA study to more reliably detect an expansion in a biologically relevant cluster. With mass cytometry, the original authors identified an interesting and robust expansion: HLA^hi^ sub-lining fibroblasts are expanded in inflamed RA samples compared with osteoarthritis (OA) controls (n = 28); however, the authors were unable to detect this known expansion in their scRNA-seq data (n = 21). We wondered whether the authors’ inability to detect this expansion was due to insufficient power, and whether we could modify their study design to increase power. Thus, we simulated realistic datasets derived from the RA fibroblast data and induced a fold change of 5 (comparable to study) in the cluster with the highest expression of *HLA* in fibroblasts (Figure S3, STAR Methods). For 20, 40, and 80 samples, we simulated datasets with unbalanced case-control proportions similar to the original study: 17 case/3 control, 34 case/6 control, and 68 case/12 control samples, respectively. The imbalance of cases and controls limited power, which was only 12% for 20 samples, 29% for 40, and 75% for 80 (Figure 5A). We then repeated these power analyses, but this time with study designs containing an equal number of cases and controls (STAR Methods). Unsurprisingly, power dramatically increased to 60% at 20 samples, 89% at 40, and a full 100% at 80 (Figure 5A). This dramatic increase is likely a result of increasing the smallest group (in this example, the number of controls), which enhanced the ability for the mixed models of MASC to detect differential abundance. Thus, we determined the number of samples needed to noticeably increase power and estimated the benefit of a study design that contains more balanced case-control proportions.

### Baseline power analyses estimate realistic power

We then performed a ‘‘baseline’’ power analysis for each dataset to illustrate power under realistic parameters (derived from input data); we used these results to contextualize the changes in power from modifying study designs. We simulated datasets with realistic cluster frequency variation and batch- and sample-level gene expression variation (STAR Methods). Each simulation contained 50 case and 50 control samples, 500 cells per sample, and four samples per batch. In each simulation, we induced a fold change difference in one uniformly random cell state.

For each experimental setting (RA, TB, and UC), we estimated power over fold changes that range from subtle to large: 1, 1.05, 1.1, 1.25, 1.5, 2, and 4, corresponding to a 0%, 5%, 10%, 25%, 50%, 100%, and 300% respective increase in a cluster’s frequency in cases versus controls. Power in the TB setting was generally greater or equal to power in the RA and UC settings (Figures 5B-5D, left, red bars). This difference likely reflects the lower estimated cluster frequency variances in the T cell-only TB data compared with the multiple cell type RA and UC data (Figure 3C).

To evaluate how estimated power relates to the size of the input dataset, we performed subsampling analyses by randomly sampling smaller datasets that were then used as input datasets (STAR Methods). For each experimental setting, the subsampled datasets generally produced lower power estimates (Figure S4). This is likely due to the input providing a less accurate estimation of realistic parameters. Notably, when subsampling the TB dataset to the pilot data (48 samples), the power estimates are similar compared with when using the full dataset as input. This suggests there exists a minimum dataset size that achieves convergent power estimates (Figure S4B).

### Removing dominant sources of variation estimates upper limits on power

Next, we wanted to estimate how intrinsic variation leading to imprecise clustering might limit power in the absence of other sources of noise. Since the TB and UC settings each have many transcriptionally similar fine-grained states within broad cell types, we predicted lower power in these settings compared with RA, whose cell states are more transcriptionally discrete.

Accordingly, we performed analyses in which we simultaneously removed three main sources of noise, which we call a minimal-noise context: we assumed no cluster frequency variation and no batch-associated or sample-associated gene expression variation, so that all noise is driven by the intrinsic (residual) gene expression variation (STAR Methods). If there were absolutely no variation, statistical tests should have 100% power to detect DA in a cluster; therefore, any reduction of power observed in the minimal-noise context is due to intrinsic gene expression variation. In all three experimental settings, we had 0% power to detect an extremely subtle fold change of 1.05, indicating that intrinsic gene expression variation limits power even in the absence of other sources of noise (Figures 5B-5D, left, teal bars). However, we estimated 100% power at fold changes as little as 1.1 and 1.25 in the RA and TB settings, respectively, showing that intrinsic gene expression variation does not limit power at these slightly higher effect sizes.

### Cluster frequency variation dominantly affects power

We next wanted to determine which of the three sources of variation we removed in the minimal-noise context had the most effect on power. Accordingly, we performed simulations in which we removed only one source of variation while leaving the other sources at realistic levels (STAR Methods).

First, and most dramatically, we removed cluster frequency variation (cf_scale_ = 0) so that each simulated sample had the same cluster frequency distribution before inducing a fold change in a cluster. Unsurprisingly, this resulted in increased power, especially in the RA and UC settings (Figures 5B-5D, right, blue bars). The higher increase in power for the RA and UC settings likely reflects the higher variation in cluster frequencies compared with those in the TB setting (Figure 3C).

Next, we removed batch-associated variation in gene expression (b_scale_ = 0) and observed minimal increases in power at most fold changes for all three experimental settings (Figures 5B-5D, right, purple bars). Finally, we removed sample-associated variation in gene expression (s_scale_ = 0) and again observed modest changes in power for each fold change in each setting (Figures 5B-5D, right, orange bars). These results suggest that cluster frequency variation has the most effect on power.

### Increasing batch-associated transcriptional variation decreases power

Batch effects can be reduced by investing time and energy into improving protocols and reproducibility; however, the value of this investment is not always clear. To quantify how much decreasing batch effects might increase power, we performed analyses in which we modulated the level of batch-associated variation (STAR Methods). We used a scaling factor (b_scale_) to scale the input-derived estimates of batch-associated variance by 0×, 0.5×, 1×, 2×, or 4×. The batch-corrected RA setting was largely resistant to high multipliers of estimated batch-associated variance, while the uncorrected batch TB and UC settings showed decreased power at higher multipliers of batch-associated variance (Figure 5E).

### Power estimations are robust to modest decreases in sequencing depth

Sequencing depth is an important property of single-cell studies because it not only affects the detection of transcripts, but also the precision with which cells are classified and, thus, power. To measure how reduced sequencing depth affects power, we first downsampled the RA dataset’s UMI counts (STAR Methods). From the original dataset (7,300 mean UMIs), we sampled datasets with 1,825, 730, 365, and 73 mean UMIs (Figure S5A). For each downsampled dataset, we applied a standard principal component analysis (PCA) pipeline. UMAP visualization suggests that the datasets with a mean of 730 UMIs or lower resulted in cluster mixing (Figure S5B). We input the PCs from each sampled dataset and the clusters obtained from the original RA dataset into scPOST.

At most fold changes, we observed minimal differences in power between the original dataset and the dataset with 1,825 mean UMIs (Figure 5F, red/dark teal). However, when downsampling to 730 mean UMIs or lower, we observed noticeable power loss at fold changes above 1.5. These results show power is robust to modest decreases in sequencing depth, but reducing to extremely low depth can eventually confound cluster classification and power.

### Multiplexed study designs can decrease batch effects and increase power

Multiplexing by running multiple samples in a single batch can reduce the number of batches (and the impact of batch effects) in a study. We sought to estimate how power is affected by reducing the number of batches (by increasing the number of samples per batch). In the TB setting, we modulated the magnitude of batch-associated variance in addition to reducing the number of batches (STAR Methods). At realistic (1×) levels of batch variance, reducing the number of batches resulted in minimal power changes (Figure S6A). In contrast, at 4× the realistic batch-associated variance, we observed that multiplexing to reduce the number of batches rescues power to levels observed at 1×. While effective, this strategy requires a small number of batches with large numbers of samples and cells, which may not be feasible in all experimental settings.

**Figure 6.**
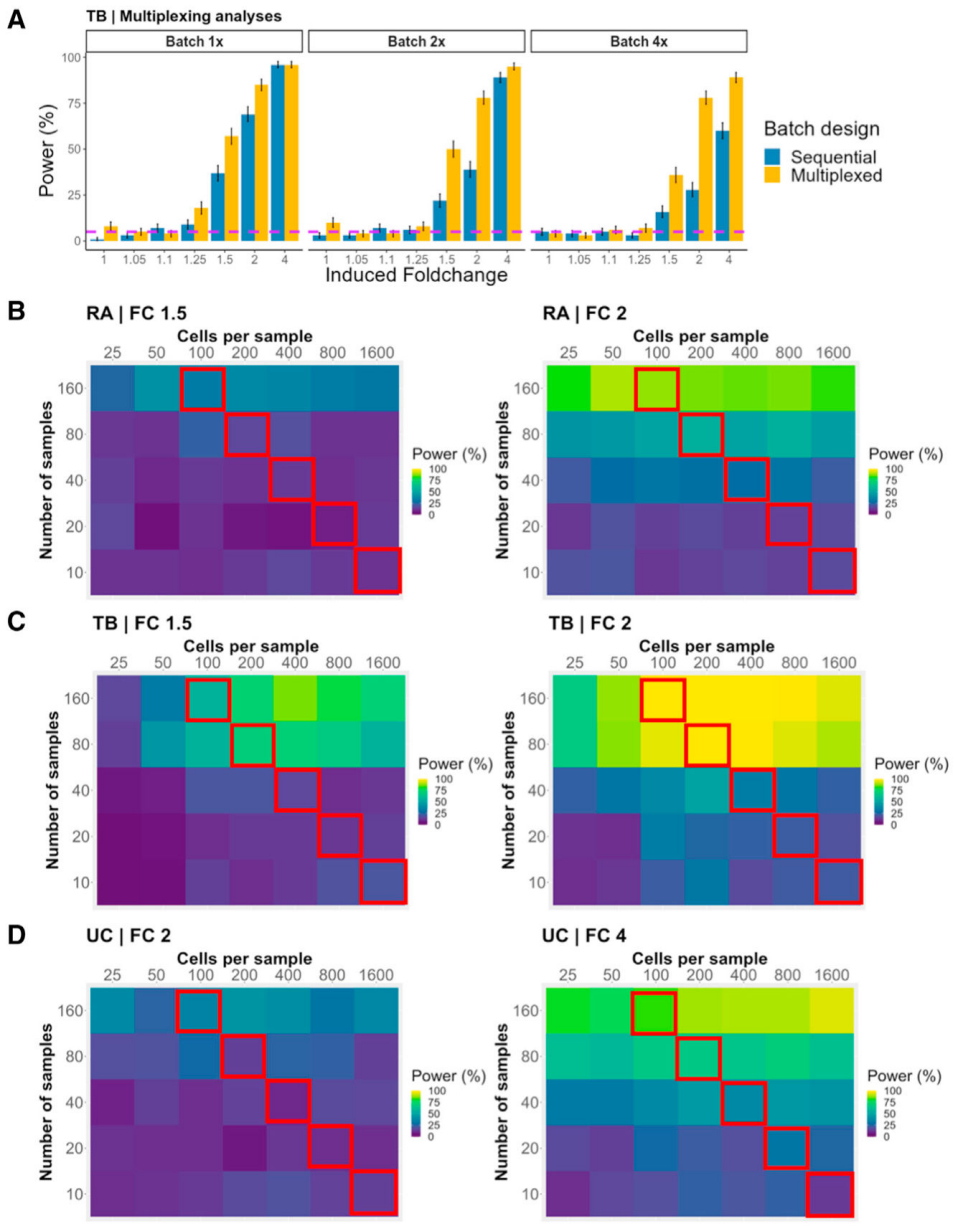
scPOST estimates benefits of multiplexing and increasing dataset size (A) Power estimates comparing a sequential versus multiplexed study design. We simulated these study designs with varying levels of batch effects: 1× (level derived from input data), 2×, and 4×. Each bar represents n = 500 simulations. Error bars represent 95% binomial proportion confidence intervals; dotted horizontal purple lines are set at 5%. (B–D) Power calculations across a range of dataset sizes at fold changes of 1.5, 2, or 4. Tiles represent n = 100 simulations. We performed simulations in the baseline context. Tiles across top left-bottom right diagonals have equivalent numbers of cells; tiles outlined in red all have 16,000 total cells.

We then investigated another multiplexing scheme that retained the same number of cells per batch and total samples, but distributed samples across multiple batches. As a control, we assessed a sequential study design with realistic parameters consisting of 100 samples (2,000 cells each) placed into 100 individual batches. We compared the control study design with a multiplexing scheme that still contained 100 samples of 2,000 cells each across 100 batches, but each batch contained 500 cells from four different samples (Figure S6B, STAR Methods). Multiplexing yielded noticeable improvements, especially at higher multipliers of batch effects (Figure 6A). These benefits are likely due to the multiplexing design enabling better estimation of batch effects on individual samples.

### Increasing the number of samples improves power more than increasing cells per sample

Collecting a large number of samples can be difficult, due to cost and limited availability. Likewise, increasing the number of cells per sample can be prohibitive due to protocol limitations and constraints on the size of each sample, leading to important tradeoffs in cost and power. Thus, we wanted to investigate whether more power is gained by increasing the number of samples versus increasing the number of cells per sample.

In each experimental setting, we simulated datasets in the baseline context while varying the number of samples and cells per sample. As an example, for simulations with 16,000 total cells (Figures 6B-6D, red outline), we simulated 10, 20, 40, 80, and 160 samples with 1,600, 800, 400, 200, and 100 cells each, respectively. In all three experimental settings, for the same total number of cells, increasing the number of samples yielded noticeably higher increases in power compared with increasing the number of cells per sample. We observed a similar pattern across all tested fold changes (Figures S7A-S7C).

**Figure 7.**
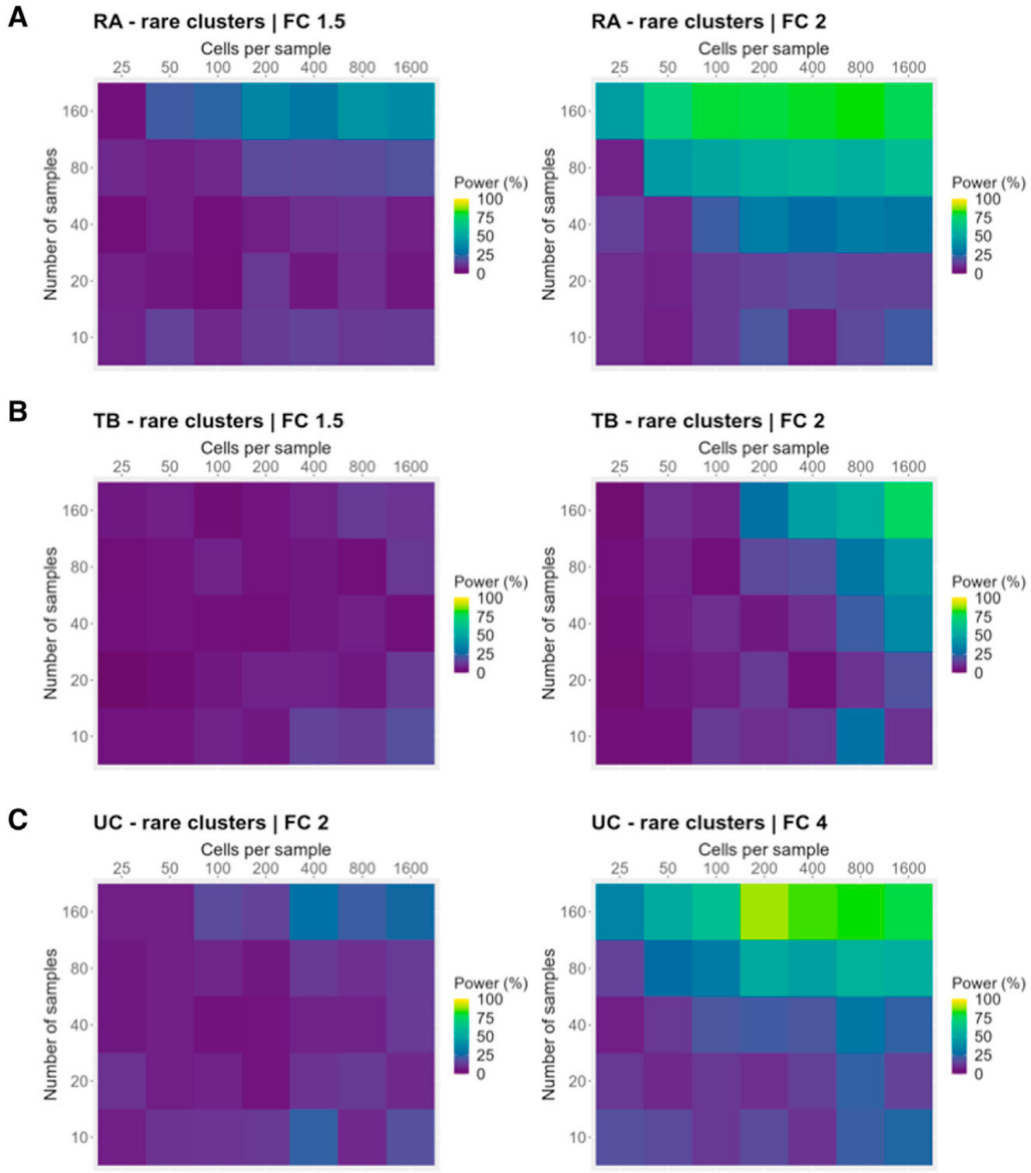
Power estimates when focusing on detecting effects in rare clusters (<1% mean frequency across samples) Power calculations for detecting effects in rare clusters across different ranges of dataset sizes at fold changes of 1.5, 2, or 4 (same fold changes as shown in Figures 6B-6D). Each tile represents power estimation from n = 100 simulations. Simulations were performed in a realistic context and frequency fold changes are induced only in original clusters that exhibited <1% mean frequency across samples.

We then performed a similar analysis focused on a scenario in which the investigator is only interested in cell types whose true abundance is rare (<1% mean frequency across samples). Rare cell types are typically harder to identify in single-cell experiments, and inaccurate frequency estimates may lead to decreased power (Figure S8A). In this scenario, increasing the number of cells per sample may be important in detecting effects. Across all three datasets, we found that when focusing on rare cell types, power was generally lower. Surprisingly, increasing the number of samples still provided similar or better power benefits compared with increasing the number of cells per sample, even when focusing on rare cell types (Figures 7A-7C). Given these results, we generally recommend that investigators focus on increasing the number of samples, although increasing the number of cells per sample may have similar power benefits in specific experimental contexts.

### Estimated power is maximized when simulated clustering structure is similar to input data clustering

In the previous simulations, we used Louvain clustering to cluster our simulated datasets before DA testing and estimating power. A key parameter of the Louvain algorithm is “resolution,” which affects the number of clusters retrieved from the algorithm. To evaluate how resolution affects the behavior and power of scPOST, we used the RA dataset as input, which featured 12 clusters retrieved from clustering at a resolution of 1.2. After simulating 100 datasets in the baseline context, we clustered each simulation over a range of resolutions and retrieved the number of clusters obtained (Figure S8B). Unsurprisingly, higher resolutions generally yielded more clusters. We then performed DA testing for each resolution in each simulation with MASC and estimated power. In general, power was similar among resolutions 0.4, 0.8, 1.2, and 2, but noticeably lower at resolutions of 0.1 and 0.2 (Figure S8C); this suggests that diluting an effect by keeping it in larger clusters can significantly decrease our power, but breaking the effect up into smaller clusters is less impactful. We further assessed power at a fold change of 2, as it was the most variable fold change between resolutions. Regardless of the resolution value, we had maximum power when the number of clusters was similar to the number of original clusters (11–14 clusters, Figure S8D).

To create a clearer picture on how clustering resolution affects the structure of simulated datasets, we used an “interpretability” metric that quantifies whether simulated cells from the original “causal” cluster (the cluster we induced a fold change into) are placed in DA clusters; interpretability quantifies how well we detect the “correct” effect (STAR Methods). Like power, interpretability scores at resolutions of 0.4, 0.8, and 1.2 were similar; however, interpretability scores for 0.1,0.2, and 2 were noticeably lower (Figure S8E). We again observed that interpretability scores tend to be maximized when the number of clusters was 11 to 14 (Figure S8F), similar to the optimized clustering of the original data. Unsurprisingly, these analyses suggest that estimated power and interpretability are maximized when the simulated clusters have similar structure compared with the input data. Overall, we recommend users try a range of resolution values.

### scPOST scales to large datasets and facilitates multi-sample, multi-cell type dataset generation

Current scRNA-seq dataset simulation methods such as SymSim (Zhang et al., 2019b) and powsimR (Vieth et al., 2017) struggle to simulate reasonably large datasets (>25,000 cells) because they simulate individual genes for each cell that requires prohibitive computational resources/time even when simulating only 2,000 genes per cell (Table S2). Current methods also lack support for simulating multi-cell type and multi-sample data, and lack estimation and generation of independent samples with cell type frequency variation. scPOST addresses these limitations and scales to large dataset generation (Table S2), which makes DA power estimation feasible.

### scPOST facilitates evaluation of alternative models and algorithms

For previous results, we used MASC, which applies mixed models that contain covariates (e.g., batch identity) to test for differential abundance. We wondered how much incorporating random effects, such as batch information, contributed to power. Thus, we used simple fixed-effects models for DA testing and compared power with MASC (STAR Methods, Figure S9A). In general, MASC provides better power than a straight fixed-effects approach, highlighting the impact of random effects.

While we analyzed simulated datasets with Louvain clustering and MASC, the generated datasets can be analyzed with alternative algorithms, especially tools that explicitly use PC information. Thus, scPOST provides an opportunity for users to explore how different algorithms perform on simulated single-cell data. For example, users may cluster the simulated datasets with an alternative clustering algorithm and estimate power. For demonstration, we used the RA dataset to simulate datasets in the baseline context and then clustered cells with the SC3 clustering algorithm (Kiselev et al., 2017) before DA testing with MASC and estimating power (STAR Methods). Estimated power was similar, regardless of whether we used the Louvain or SC3 clustering algorithm (Figures S9B and S9C).

A new class of DA tests focuses on using neighborhoods as an alternative to clusters. Milo (Dann et al., 2020) is a DA neighborhood test that uses a dataset’s PCs as input to find neighborhoods and calculates a false discovery rate (FDR) value for each neighborhood to determine whether that neighborhood is DA between conditions. To evaluate Milo’s performance, we used the RA dataset to simulate datasets in the baseline context and then used Milo for DA testing instead of MASC (STAR Methods). Notably, we detected significant DA neighborhoods even when no effect was induced at a fold change of 1 (Figure S9D). Even when cf_scale_ is equal to 0 (meaning the fold changes between conditions are exact), we detected DA neighborhoods at a higher rate than expected at the FDR <5% level (Figure S9E). Notably, while power generally did not vary with effect size at cf_scale_ = 1, the percentage of DA neighborhoods increased as effect size increased (Figure S9F). These results warn that significance values at the neighborhood level may not be well calibrated and should be contextualized by a more global metric that relates to how many DA neighborhoods are present in the data.

scPOST uses a cluster-based framework, as parameter estimation and dataset generation occur at the cluster level. Since Milo uses neighborhoods, it is possible that Milo’s performance is affected by using a cluster-based input. Hence, we modified the RA input to become a neighborhood-based input by instead labeling cells with the neighborhood they belonged to as designated by Milo (STAR Methods). Neighborhoods that contained <5 cells were excluded from the input. Even with a neighborhood-based input, power estimates remained similar, with a higher rate of positive detections than expected at a fold change of 1 (Figure S9G).

## DISCUSSION

Here, we demonstrate the utility of scPOST by simulating thousands of large single-cell datasets to investigate how different study design choices affect power to detect differential abundance between conditions, thus informing optimal allocation of limited resources, e.g., cost of patient samples and single-cell assays. scPOST is particularly useful because it estimates power from a user-defined prototype dataset, which may be a pilot or public dataset, such as from the Human Cell Atlas (Regev et al., 2017), that reflects the planned experimental setting.

In a powerful use case, we used scPOST to determine how to expand the 21-sample RA study to more robustly detect an expansion of *HLA*^*hl*^ fibroblasts. We found we could significantly increase power by increasing the number of samples and adopting a study design with a balanced proportion of case and control samples. Given that we and others find that shallower sequencing or assaying fewer cells per sample does not generally compromise power (Pollen et al., 2014; Mandric et al., 2020), it is more effective for investigators to allocate these resources to obtaining more samples.

We observed several patterns across three diverse experimental conditions. We consistently found that three factors substantially influenced overall power: the number of independent samples, a cluster’s frequency variation across samples, and the magnitude of batch effects. In contrast, we found that acquiring more cells per sample and deeper sequencing had more modest effects on power.

Minimizing batch effects can result in higher power and increased accuracy in the identification of cell states. We observed that a multiplexing scheme that splits samples across multiple batches can provide significant benefits to power by reducing batch effects. If it is more difficult for a study to obtain more samples, but the samples are big enough to be split into multiple batches, using this multiplexing scheme may be a way to increase the study’s power.

The datasets generated by scPOST are easily compatible with tools that explicitly use PCs as input. Thus, users may explore how alternative clustering algorithms, differential abundance tests, or association testing schemes perform on simulated single-cell data as parameters vary (such as increasing the number of samples and/or cells per sample).

We envision that scPOST will be applied to representative public datasets or pilot studies in order to predict power of future studies. Alternatively, our tool can be used retrospectively to determine the range of effect sizes that a completed study is actually powered to detect. As single-cell studies become larger and shift toward comparing conditions, we expect scPOST to be broadly useful for investigators in many experimental settings for defining optimal study designs.

### Limitations of the study

We chose to simulate gene expression as PC coordinates; this precludes these datasets from directly being analyzed by tools that require raw gene expression data (although PC loadings allow us to work backward to retrieve simulated gene expression). Nevertheless, scPOST allows users to output the simulated datasets, which can be analyzed with tools that use PC coordinates or graphs derived from PC coordinates, such as alternative clustering algorithms (SC3), alternative differential abundance algorithms (Milo), or trajectory analysis (Haghverdi et al., 2016; Qiu et al., 2017). With newer multimodal technologies such as CITE-seq becoming more available, analyses of these new data types may include dimensionality reduction with alternative methods, such as canonical correlation analysis (Stuart and Satija, 2019) and nonlinear embeddings (Gayoso et al., 2019, 2020). Simulating new types of data in the context of these alternative tools, such as simulating canonical variate coordinates instead of PC coordinates, represents a possible extension of scPOST. Another potential extension is associating cluster frequencies with alternative variables beyond case-control status, including continuous variables (e.g., polygenic risk score).

We showed that the size of the prototype dataset may affect power estimation, especially when the number of samples or cells is small. Our benchmarking strategy emphasizes clusters found in the prototype data; thus, if a study’s focus is on a rare cell type that is potentially missing in a small prototype dataset, then power estimates may not be well-calibrated.

The current scPOST pipeline focuses on estimating power to detect differential abundance. Alternative effects that investigators commonly search for include differential gene expression between conditions, or shifts in trajectories. To explicitly test for these effects, scPOST would require further extension so that simulated data include these effects.

## STAR★METHODS

### RESOURCE AVAILABILITY

#### Lead contact

Further information and requests for data should be directed to and will be fulfilled by the lead contact, Soumya Raychaudhuri (soumya@broadinstitute.org).

#### Materials availability

This study did not generate new unique reagents.

#### Data and code availability

All datasets analyzed in this paper are publicly available. Processed scRNA-seq data for the RA dataset is available at ImmPort accession SDY998, and the raw scRNA-seq is available at dbGaP accession phs001457.v1.p1. Processed scRNA-seq data for the UC dataset is available at Single-Cell Portal: SCP259. We provide processed metadata tables (including cell state cluster assignments) and gene expression matrices for the RA and UC datasets at https://github.com/immunogenomics/scpost. The memory T cell CITE-seq data from the TB dataset is available at GEO accession GSE158769. Links for each dataset are included in the key resources table.

An R implementation of the scPOST framework (along with input prototype data structures from the RA and UC datasets) is provided at https://github.com/immunogenomics/scpost. scPOST was deposited to Zenodo at https://doi.org/10.5281/zenodo.5573126.

Any additional information required to reanalyze the data reported in this paper is available from the lead contact upon request.

### METHOD DETAILS

#### Overview of single-cell POwer Simulation Tool (scPOST)

The goal of scPOST is to provide a flexible tool to simulate single-cell datasets that approximate an experimental context. scPOST uses input prototype datasets (e.g. a public dataset or pilot dataset) to estimate characteristic qualities of an experimental setting, such as the variation in cell state (cluster) frequencies across samples and the gene expression structure of cells likely to be measured in the setting. By modifying the study design of the generated datasets, investigators may use scPOST to predict how specific study design choices might affect the analysis results of a single-cell experiment. We envision scPOST will be helpful for investigators in planning the design of their studies so that they may maximize their power to detect effects, such as differential abundance (DA) of a cell state between conditions (e.g. expansion of a cell state in case samples versus control samples).

Here, we present scPOST as comprising three steps: (1) parameter estimation that takes a prototype dataset as input, (2) simulation of a new single-cell dataset based on the estimated parameters, and (3) power calculations from performing DA testing with Mixed effects Association of Single Cells (MASC). We note that users may stop after the second step, and instead perform other analyses on the simulated datasets. The user is able to customize the construction of the simulated dataset in several ways including: the number of total cells, the number of total samples, the number of cells per sample, the number of batches, multiplexing structure, and the magnitude of noise that contributes to the variation of gene expression or cluster frequencies. By modifying how the simulated data is generated, investigators may use scPOST to estimate how changes in study design affect their ability to detect effects.

Currently, scPOST can produce two different types of output: (1) simulated datasets in the form of each cell’s meta information and principal component coordinates (2) power calculations from DA testing. Most results presented in this paper are power calculations from DA testing, but users may instead retrieve the simulated datasets and perform alternative analyses, a procedure we utilize for the SC3 and Milo analyses. As simulated datasets are generated in the form of principal components, the most compatible analyses are those that utilize principal components. Implementations of scPOST are available as part of an R package at https://github.com/ immunogenomics/scpost, along with several of the input prototype datasets showcased in this paper. The following sections explain the procedures used in the scPOST framework. We begin with how scPOST simulates single-cell datasets, and then proceed into DA testing with MASC and power estimation. We conclude with procedural details about specific analyses presented in the paper.

#### Step 1: Parameter estimation

We estimate and simulate gene expression in the form of principal components (PCs). In the context of scRNA-seq analysis, PCs capture gene expression covariation and are frequently used to summarize high-dimensional scRNA-seq data. To generate a new single-cell dataset *S* in the form of a cell by PC matrix, we estimate several key parameters from the original input prototype dataset. The PC embeddings (received from PCA) for each cell, as well as their cluster classifications (established by Louvain clustering) are used as input for parameter estimation.

##### Using linear mixed models to estimate gene expression variation summarized in principal component space

We focus on variation in PC space, which represents variation in gene expression. We perform the following procedures in a cluster-specific manner because variance in PC space can differ between cells in different clusters; differential variances are often modeled and corrected for in batch-correction algorithms. In the parameter estimation step, we focus on each cluster’s variance along each PC. We deconvolute this total variance into different sources (batch, sample, or residual) with linear mixed effects models. Here, we explicitly estimate contributions that batch and sample identity have on gene expression variation, because batch and/or sample identity are: (1) covariates that are commonly thought of as contributing to variation, (2) tend to be covariates that are corrected for in scRNA-seq analyses, (3) almost always present in metadata. We note that this process is generalizable to many covariates, and models can be fit with an alternative set of covariates (e.g. only fitting a model that explicitly estimates variation contributed from sample identity). If users choose to model a different set of covariates, they should also augment the estimation and simulation steps to include how they expect the new/different covariates to affect gene expression.

To deconvolute cluster-specific variance in PC space, we fit linear mixed effects models of the form:

(Equation 1) Y=Xβ+Zu+ε

Where the unconditional distribution of random effects designated by *u* are:

(Equation 2) $$u∼N(0,\tau^{2})$$

Beta represents an unknown vector describing fixed effects. In our model, beta empirically estimates the PC centroid for a cluster. Epsilon represents unknown vectors of random errors (residuals). In our model, epsilon empirically estimates the remaining variation not explicitly captured by the batch and sample covariates that were included as random effects in the model. The *τ*^2^ parameter that is estimated for each random effect (in this case, batch and sample) is an empirical estimate of the effect’s variance contribution to the response variable. Again, we assume that each cluster has different batch-associated and sample-associated variation. Thus, for each cluster *k*, we fit the following formula for each PC (default of 20 PCs) with the R function “lmer” from the “lme4” package:

(Equation 3) PC∼1+(1∣batch)+(1∣sample)

Because we fit random models for each PC for each cluster, we fit *k* × *nPC* models, where nPC is the number of PCs. After fitting, we extract the following elements from each model:

μ_k_: The fixed effect intercept of a model, representing the unconditional mean value in PC space. The mean value for all PCs of a cluster represents its centroid.

Σ_B_: The estimated *τ*^2^ values for the batch random effect fitted for each PC, representing the contribution batch identity has on gene expression variation in a cluster.

Σ_S_: The estimated *τ*^2^ values for the sample random effect fitted for each PC, representing the contribution sample identity has on gene expression variation in a cluster.

Σ_C_: The estimated residuals fitted for each PC, representing the remaining variation not explicitly estimated from our covariates. We interpret this value as variation “intrinsic” to the cluster, but some of this variation may contain variance associated with other covariates we did not fit.

For each cluster, we enforce Σ_B_ and Σ_S_ to be diagonal matrices where the diagonal elements are the estimated *τ*^2^ values for batch or sample respectively. The residual values Σ_C_ are placed into an *nPC x nPC* variance-covariance matrix for each cluster.

##### Estimating cluster frequency variation

For each cluster, we estimate its mean frequency and covariation across samples. These variances can be relatively large, especially if the samples are from humans. The observed cluster frequency (CF) variation is likely a result of: (1) the differences between the true distribution of cell states from the sample’s origin and (2) technical sampling variation induced by obtaining and processing the samples. Here, we do not distinguish between these different sources of variation.

To calculate the mean and variance of cluster frequencies in a dataset, we first count the frequency of each cluster in each sample and increment these frequencies by a pseudo-count of 1. Within each sample, the frequencies are transformed into proportions by dividing each frequency by the total number of cells within that sample. The mean of these proportions across all samples is represented by μ_cf_.

During dataset generation (**Step 2**), we sample CF distributions in log-space. Thus, μ_cf_ is transformed into log-space. Furthermore, the aforementioned proportions are then log-transformed, which we then use to calculate a variance-covariance matrix of the cluster frequencies Σ_cf_ in log-space.

#### Step 2: Generating a single-cell dataset

In our simulations, we use an input prototype dataset *X* to generate a new single-cell dataset *S* in the form of a cell by PC coordinate matrix. As part of dataset generation, we assign each single-cell the following meta information: (1) batch identity, (2) sample identity, and (3) the original cluster assignment, which is used to generate its PC coordinates. Note, the original cluster assignments may differ from clustering assignments produced in downstream analysis (which is performed in **Step 3**). Overall, we simulate datasets based on two general aspects of variation in a single-cell dataset: the variation in cluster frequencies across samples, and the variation in gene expression.

##### Simulating cell state frequency distributions

The power to detect differential abundance (DA) in an experiment is dependent on the frequency distributions across the samples. Thus, it is important for simulations to generate cluster frequency (CF) distributions for each sample. scPOST allows users to generate CF distributions that reflect the observed CF variation in a specific experimental setting, as well as increase or decrease the simulated levels of CF variations. As described in the Section "Estimating cluster frequency variation", we empirically estimate a *k*-length mean CF distribution μ_cf_ (whose elements contain the observed mean frequency of a cluster across all of the samples in the dataset) and its corresponding variance-covariance matrix Σ_cf_.

When sampling CF distributions, we sample in log-space so that we do not sample negative frequencies. Thus, we first transform μ_cf_ and Σ_cf_ into log-space. For each simulated sample, we generate their CF distribution from a multivariate normal distribution parameterized by mean μ_cf_ and covariance Σ_cf(both now in log-space);_ we allow users to scale the amount of CF covariation that is generated via a scaling parameter cf_scale_. If cf_scale_ is set to 0, the simulated samples will all have the exact same CF distributions. The sampled *k*-length vector is then transformed back into linear space. A CF distribution *F* is thus distributed as:

(Equation 4) $$F∼e^{N(\mu_{cf},\;\Sigma_{cf}\;*\;cf_{scale})}$$

Finally, each value in *F* is divided by the sum of all values in *F*to create a probability distribution. Each sample’s generated CF distribution is used to determine how many of its cells are assigned to each cluster. Samples are pre-assigned to batches based on the user-defined batch structure (e.g. 16 samples split into four batches, with four samples per batch). We provide functions that help users split samples into batches based on the R function “split”.

##### Inducing a fold change difference in a cluster between conditions

Here, we define a fold change in a cluster as the ratio of cells in one condition compared to the other (e.g. a fold change of 4 means the number of case cells in a cluster is 4× compared to the number of control cells in a cluster). In our simulations, we induce a user-defined fold change difference in one cluster, which is the differential abundance we wish to detect. To induce a fold change difference in a cluster between conditions, we alter all of the CF distributions generated for all samples in one condition. Here, we alter the case samples. Given an initial generated CF distribution, we first scale the frequency of the selected cluster by the magnitude of the fold change we wish to induce, which gives us the new frequency for the cluster of interest and some remaining probability mass. The frequency for the cluster is capped at 1, which would mean all cells from that sample come from this cluster. The frequencies for the remaining clusters are then divided by their sum (the sum of the frequencies of the remaining clusters), and then scaled by the remaining probability mass. If there is no CF variation (cf_scale_ = 0), then the observed fold change will be exact. With CF variation, the observed fold change will vary around the intended effect size.

##### Representing single cells in PC coordinates

Principal components derived from principal component analysis (PCA) on a cell by gene matrix, are summaries of gene expression and represent data structure in the context of covariation between genes. PCA is commonly used in dimensionality reduction pipelines for single-cell analyses. The PC embeddings (values) for each cell represent its location in PC space and its gene expression signature relative to other cells. Cells closer to each other in PC space generally have more similar gene expression profiles than cells further away from each other, which is a consequence of PC coordinates encoding covariation structure between multiple genes. We estimate variation in PC space in parameter estimation (**Step 1**), and we generate PC coordinates for each simulated cell (**Step 2**). The generated PC locations for our simulated cells *L* can then be used for downstream analysis.

##### Simulating PC coordinates for a cell state

Variation in gene expression can influence our ability to detect differential abundance, as high variation may result in misclassification of cell states via clustering algorithms. Our simulation approach assumes that each cluster has different levels of variation, which is clear from variance estimation. Furthermore, we assume that the gene expression of each cell state is affected by batch or sample effects (derived from the batch the cell was run in or the sample origin respectively) differently.

For simulated cells, we assign initial cluster identities that are analogous to the clusters in the original input dataset *X*; these assignments are derived from generated CF distributions that we defined in Equation 4. We generate PC coordinates based on the simulated cell’s assigned cluster identity. If *c* is the number of cells in a simulated dataset and nPC is the number of PC dimensions we simulate, we simulate a matrix of PC coordinates whose dimensions are *c* × *nPC*.

For each cluster, we assume that its PC coordinates are distributed around some nPC-dimensional centroid μ with covariance Σ_C_. In our simplest generative model without batch or sample effects, we simulate an nPC-dimensional vector of PC coordinate for each cell based on its assigned cluster. To generate *L*, we sample from a multivariate normal distribution parameterized with mean μ and covariance Σ_C_, giving *L* the following distribution:

(Equation 5) $$L∼N(\mu ,\Sigma_{C})$$

##### Simulating technical effects (batch and sample-level effects)

Here, we use linear mixed models to explicitly model the magnitude of batch-associated variation in PC space and sample-associated variation in PC space. These models allow us to estimate how much variation that batch identity and sample identity contribute to a specific model’s total variation in PC space. As we assume the contribution of batch and sample effects is cluster-specific, we generate a batch/sample effect for each cluster we simulate.

We generate batch and sample-level effects by using the parameter estimates Σ_B_ and Σ_S_ that we retrieved from **Step 1** (Equation 3). These generated effects take the form of linear shifts that are summed with their respective centroid. We generate batch linear shifts with a multivariate normal distribution with mean 0 and covariance Σ_B_, which represents the cluster-specific variance of cells in PC space contributed from batch effects. Similarly, we generate sample linear shifts with a multivariate normal distribution with mean 0 and covariance Σ_S_, which represents the cluster-specific variance of cells in PC space contributed from sample effects.

In order to modulate the magnitude of these batch and sample linear shifts (and thus the magnitude of the batch/sample effects), we use scale factors, b_scale_ and s_scale_ respectively. If b_scale_ is set to 0, there will be no batch linear shift (equivalent to 0 batch-associated effects in the simulated dataset). The same is true for s_scale_. These scale factors are used to modulate the covariance of the multivariate normal distributions we sample from. Thus, our simulated batch/sample linear shifts for batch *m* or sample *d* respectively have the distributions:

(Equation 6) $$b_{m}∼N(0,\;\Sigma_{B}*b_{scale})$$

(Equation 7) $$s_{d}∼N(0,\;\Sigma_{S}*s_{scale})$$

Each cluster’s batch and sample linear shifts are summed with its respective cell state centroid in order to create an adjusted centroid that is dependent on cluster assignment, batch identity, and sample identity. Thus, a cluster’s adjusted centroid for cells from batch *m* and sample *d*, μ′_md_, is formulated as:

(Equation 8) $$\mu_{\;md}^{\prime }=\mu +b_{m}+s_{d}$$

In our generative model that incorporates batch and sample-level effects on gene expression, we generate a cell’s PC coordinates based on its cluster’s adjusted centroid and its respective cell state covariance Σ_C_. Thus, the PC locations for cells from batch *m* and sample *d*, *L_md_*, are distributed as:

(Equation 9) $$L_{md}∼N(\mu_{\;md}^{\prime },\Sigma_{C})$$

#### Step 3: Power estimation from differential abundance testing

##### Testing clusters for differential abundance (DA) with MASC

The output of **Step 2** is the PC coordinates of a generated single-cell dataset. For DA testing, we first generate new cluster labels for each cell (which are based on the generated PCs) by creating a shared nearest neighbor (SNN) graph (k = 30) and using Louvain’s method of community detection (resolution controlled by the user). For most analyses, we used the same resolution that was used for the original input prototype dataset. From this procedure, we obtain new cluster assignments for each of our simulated cells.

We test each cluster for DA between two conditions, which we refer to as case vs. control. A simple DA test would be to utilize Welch’s unequal variance t-test to test whether a cluster’s mean frequency in cases is significantly different than the same cluster’s mean frequency in controls. Notably, this procedure utilizes sample-level information, but not information provided by other covariates (such as batch). The inability to control for more covariates may lead to inflated p values. Thus, we use an alternative method called Mixed effects Association testing for Single Cells (MASC), which is a DA test that utilizes logistic mixed effects models to test whether a cluster’s identity is associated with its condition status. Importantly, MASC allows the user to control for potentially confounding single-cell covariates and has well-calibrated type 1 error. For all of our results, the null and full models used as input for MASC are as follows:

(Equation 10) Null:clusterIdentity∼1+(1∣batch)+(1∣sample)

(Equation 11) Full:clusterIdentity∼condition+(1∣batch)+(1∣sample)

Where *clusterIdentity* refers to the cluster assignments obtained from clustering the simulated dataset, *condition* refers to the category the simulated cell belongs to (e.g. case or control), *batch* refers to the batch the cell belongs to, and *sample* refers to the sample the cell belongs to.

For most analyses presented in this paper, we performed many simulations. In each simulation, we randomly chose a cluster to induce a differential abundance in. The power results we report are an aggregate of all simulations, meaning that the power represents an “overall” power across all cell states in the experimental setting. We allow users to focus on specific clusters by either choosing only a specific cluster to induce an effect in, or by stratifying their power results by cluster.

##### Power estimation

For each simulation, we test all clusters for differential abundance. Here, we considered a simulation to have successful detection if at least one cluster had DA between conditions. Significance is defined by the Bonferroni-corrected threshold of p < 0.05/*k*, where *k* is the number of clusters tested in the simulation. We note that it is often the case that the cluster we induce a fold change into contributes to one of these clusters, but we do not require it to. This is because when the number of cells in a sample is fixed, changing the frequency of a cluster in a sample inherently changes the frequency of other clusters in the sample. Furthermore, cluster frequencies can covary with others, which could potentially result in a covarying cluster exhibiting DA, even though we did not induce a fold change into the cells comprising the cluster. To quantify how much we are detecting the “correct” effect, we utilize an interpretability metric that we define in the next section.

For power estimation, we performed many simulations (either 100 or 500) for each combination of parameters and defined power as the number of simulations in which we successfully detected DA. 50% power means we successfully detected DA in half of the simulations. The error bars in presented power plots are 95% binomial proportion confidence intervals.

##### Quantifying interpretability of a simulation

It is possible in a simulation for us to detect a cluster which contains cells with an originally assigned cluster identity that is not the cluster we induced a fold change in. At the beginning of the data generation step, each cell is assigned a sample, batch, and an “original” cluster identity. After the dataset is generated, we then re-cluster the simulated dataset, and each cell is also given a “new” cluster identity. In this framework, we define the “causal” cluster as the “original” cluster that we induce a fold change in. With these definitions, we look at each “new” cluster and determine how many cells it contains that had an “original” cluster identity that was the “causal” cluster. Thus, for each simulation, we quantify how much we are detecting the “correct” effect in a simulation by calculating an interpretability score with the following procedure:

1. Determine if the simulation contains significantly DA clusters. If there are no DA clusters, we set interpretability to 0. If there are DA clusters, we proceed to step 2.
2. Give each cell in a simulation a value of 1 or 0 based on whether it was originally assigned to the cluster we induced an effect into.
3. Give each cell in a simulation a value of 1 or 0 based on whether it belongs to a simulated cluster that we detected differential abundance in.
4. Calculate the correlation between the values generated in 2 and 3.

Thus, interpretability quantifies a combination of: (1) how many cells that should end up in a simulated DA cluster actually ended up in a DA cluster, and (2) how many cells that should not have ended up in a DA cluster ended up in a DA cluster. If cells from the original causal cluster do not end up in DA clusters, interpretability tends towards zero. If cells from the original causal cluster end up in DA clusters and cells that are not from the original causal cluster do not end up in DA clusters, interpretability tends toward 1. An overall interpretability score is calculated for each simulation in the clustering analyses presented in Figure S8.

#### Analysis details

As outlined in Table S1, the rheumatoid arthritis (RA), tuberculosis (TB), and ulcerative colitis (UC) datasets were obtained from three separate studies with diverse settings. The scRNA-seq RA data were derived from synovial tissue obtained from joint replacement procedures or ultrasound-guided biopsies. The scRNA-seq TB data were derived from peripheral blood mononuclear cells (PBMCs) in blood. The scRNA-seq UC data were derived from intestinal tissue obtained from biopsies.

##### Pre-processing scRNA-seq data

We applied standard scRNA-seq pre-processing steps for the RA, TB, and UC datasets independently. We removed cells whose total reads consisted of >20% mitochondrial reads. For the TB dataset, we removed the small number of gamma delta T cells so that only memory T cells remained. For the UC dataset, we removed cells from uninflamed colitis samples taken from case donors so that we only used cells from healthy colitis samples taken from healthy donors and inflamed colitis samples taken from case donors. The final number of cells used for our analyses is 5,265, 496,517, and 23,229 cells for the RA, TB, and UC datasets respectively.

We then applied standard log(CP10K + 1) normalization for gene counts using a scale factor of 10,000; for the RA dataset, we used log2 to mimic the original author’s analysis, while we used natural log for the TB and UC datasets. For each dataset, we found the top 2,000 variable genes using the “vst” option of the Seurat function FindVariableFeatures and then z-scored the genes.

##### Principal components analysis and batch correction

We performed PCA with the R function “rsvd” from the “rsvd” package. As is standard, we weighted the resulting eigenvectors by the calculated eigenvalues to retrieve the PC embeddings. For downstream analyses, we utilized the top 20 PCs. For reproducibility, we provide the PC embedding matrices and the meta tables that we used for the RA and UC datasets in the form of objects included in the scPOST package (ra_HarmObj and uc_Obj respectively).

We applied batch correction to the RA dataset with the Harmony batch-correction algorithm in order to gain insight into how scPOST performs on data with minimal batch effects (e.g. what deconvolution of variation looks like when there is minimal batch effects). We used the HarmonyMatrix function from the “harmony” package with default parameters except for the following: var-s_use = c(“plate”, “sample”), do_pca = FALSE, and npcs = 20.

##### Clustering algorithms

For the Louvain clustering algorithm, we first built a shared nearest-neighbor (SNN) graph (k = 30) from PC embeddings, and then using Louvain’s method for community detection. We used resolutions of 1.2, 2.0, and 0.4 on the original RA, TB, and UC datasets respectively, as these produced similar clustering results to the original studies. For most analyses in this paper, we clustered the simulated datasets at the same resolution as the input dataset (e.g. we simulated RA datasets at the same 1.2 resolution as the original RA dataset). For the broader cluster analysis, we varied the resolution parameter value.

For the SC3 clustering algorithm, we utilized the “SC3” Bioconductor package. After inputting our simulated RA dataset PCs into single-cell experiment (SCE) objects, we input the SCE objects into the “sc3” function. We set the number of “ks” to be the number of original RA clusters, as well as an increment and decrement of that value (11–13). We set “biology = FALSE” and “gene_filter = FALSE”.

##### Fitting linear models

To fit our linear mixed models, we used the “lmer” function from the “lme4” package with the “nloptwrap” optimizer.

##### Parameter estimation from input datasets

To estimate parameters as described in **Step 1**, we utilized the “estimateFreqVar” and “estimatePCVar” functions from the scPOST package. The input for each independent dataset included the PCA embeddings, as well as the metadata (which contained clustering assignments for each cell).

##### Simulating realistic datasets

To simulate the realistic datasets showcased in Figures 4 and S2, we utilized unmodified estimated parameters from **Step 1**. This means when simulating with scPOST, we set the parameters “b_scale_ = 1”, “s_scale_ = 1”, and “cf_scale_ = 1” so that the simulation step simply used the estimated parameters for batch-associated variation, sample-associated variation, and cluster frequency variation. The number of simulated cells for the realistic dataset in the RA, TB, and UC settings were 5,250, 496,503, and 235,200 cells, respectively.

##### Dimensionality reduction with UMAP or tSNE

To perform dimensionality reduction for visualizing PC structure, we used either uniform manifold approximation and projection (UMAP) or t-distributed stochastic neighborhood embedding (tSNE). For each dataset, we input the PCs from both the original dataset and a simulated realistic dataset into either algorithm. For UMAP, we used the “umap” function from the “uwot” package with default parameters. For tSNE, we utilized the “Rtsne” function from the “Rtsne” package with default parameters.

##### Calculating silhouette scores

To calculate silhouette scores for the cells in a dataset, we used the “silhouette” function from the “cluster” package with default parameters. We note that we are not using the average silhouette score to evaluate quality of clustering, but instead looking at the distribution of silhouette scores within each cluster to observe how well the cells within each cluster belong to that cluster. Similarity in distributions between the real and simulated data suggest that they have similar clustering structure.

##### HLA^hi^ *fibroblast power analyses*

In these analyses, we focused only on the 1,884 fibroblasts contained in the RA dataset. With only these fibroblasts, we performed the same PCA and clustering steps we applied to the other datasets. We provide the PCA embedding matrix and meta table for these fibroblasts in the object, ra_FibObj.

For each generated dataset, we assigned samples (250 cells each) into batches equally so that each batch contained 4 samples each (accomplished with our provided “distribSamples” function). We induced a fold change of 5 in the *HLA*^*hi*^ fibroblast cluster (which is cluster 0, index 1 in the fibroblast-only data; cluster 3, index 4 in the whole RA data), and set b_scale_, s_scale_, and cf_scale_ all equal to 1. For the unbalanced study designs, we set the following sample splits: 17case/3control for 20 samples, 34case/6control for 40 samples, and 68case/12control for 80 samples. For balanced study designs, we set the following sample splits: 10case/10control for 20 samples, 20case/20control for 40 samples, and 40case/40control for 80 samples. We ran 500 simulations for each combination of parameters.

##### Baseline power analyses

For the baseline power analyses (realistic), we assigned 100 (50 case, 50 control) samples (500 cells each) into 25 batches equally with the “distribSamples” function so that each batch contained 4 samples each. We induced the following fold change in case samples: 1 (no fold change), 1.05, 1.1, 1.25, 1.5, 2, and 4. For each simulation, we induced fold change into a randomly chosen cluster. We set b_scale_, s_scale_, and cf_scale_ all equal to 1. We ran 500 simulations for each combination of parameters.

##### Subsampling analyses

To sub-sample each independent dataset, we randomly sampled 10 samples from the dataset. For the TB setting, we also used the pilot dataset (48 samples) as comparison. For each subsampled dataset, we input the PCA results and metadata into scPOST. For dataset generation, we utilized the same study design as the baseline power analyses.

##### Minimal-noise power analyses

For the minimal-noise context, we used the exact same study design as the baseline analyses, but we set b_scale_, s_scale_, and cf_scale_ all equal to 0. We ran 500 simulations for each combination of parameters.

##### Variance-removal analyses

For the singular variance-removal analyses, we used identical study design parameters to the baseline analyses. However, for the cluster frequency variation removal (No CF var), we set cf_scale_ to 0 (but kept b_scale_ and s_scale_ equal to 1). For the batch variation removal (No Batch Var), we set only b_scale_ to 0. For the sample variation removal (No Sample Var), we set only s_scale_ to 0. We ran 500 simulations for each combination of parameters.

##### Modulating levels of batch effects

For these analyses, we used the same study design as the baseline analyses. However, we tested a range of b_scale_ values: 0, 0.5, 1, 2, and 4. The results we reported are for an induced fold change of 1.5 in the RA and TB settings and an induced fold change of 4 in the UC setting. We ran 500 simulations for each combination of parameters.

##### Binomial downsampling of UMIs from the RA dataset

To downsample the RA dataset, we took the raw UMI data from the RA dataset and performed random binomial draws for each UMI count using the “rbinom” function. The binomial distribution in this model is parameterized by *n* and *p*, where *n* is equal to the original UMI count, and *p* is equal to the percentage of UMIs we wanted to sample (e.g. 25%) so that we approach a specific number of mean UMIs. We downsampled the RA dataset to create datasets that have approximately 1825, 730, 365, or 73 mean UMIs. For each downsampled dataset, we applied the same pre-processing, PCA, and clustering steps as the original RA dataset. We used the PCA results for each downsampled dataset and the original RA dataset’s clustering assignments as input for **Step 2**.

For dataset generation, we used the same study design as the baseline analyses. We ran 500 simulations for each combination of parameters.

##### Analyses with fewer simulated batches (increased samples per batch)

For these analyses, we used almost the same parameters as the baseline analyses. However, we varied the number of samples that were placed into each batch, so that the studies had 2 (50 samples per batch), 5 (20 samples per batch), 10, 15, or 25 batches overall. We utilized the “distribSamples” function in the scPOST package to place a specific number of cases and controls into a specific number of batches. We also varied the values of b_scale_: 0, 0.5, 1, 2, and 4 to evaluate how much batch-associated variation affects power across the different number of batches. We ran 500 simulations for each combination of parameters.

##### Multiplexing analyses

For the sequential study design, we assigned 100 samples (2000 cells each) into 100 batches using the provided “distribSamplePerBatch” function so that each batch contained cells from only one sample (2000 cells per batch). We induced fold changes into a randomly chosen cluster, and we set b_scale_, s_scale_, and cf_scale_ all equal to 1.

For the multiplexing study design, we split 100 samples (2000 cells each) into four equally sized subsamples (500 cells each). We then assigned these subsamples into 100 batches using the provided “distribSplitSample” function so that each batch contained cells from four different subsamples (2000 cells per batch). We induced fold changes into a randomly chosen cluster, and we set b_scale_, s_scale_, and cf_scale_ all equal to 1. We ran 500 simulations for each combination of parameters for both analyses.

##### Increasing the total number of samples versus number of cells per sample

For these analyses, we used the same study design as the baseline analyses, but we varied the total number of samples (10, 20, 40, 80, or 160) and the number of cells per sample (25, 50, 100, 200, 400, 800, and 1600). For the main figures, we show results for fold changes of 1.5 and 2 for the RA and TB settings, and results for fold changes of 2 and 4 for the UC setting. We provide power curves for the rest of the fold changes in supplementary figures. We ran 100 simulations for each combination of parameters.

For analyses that focused on rare clusters, we performed the same analyses, but we only induced a fold change in clusters with a mean frequency <1% across samples.

##### Broad clustering analyses

For the broad clustering analysis presented in Figure S8, we utilized the RA dataset as input. We simulated datasets in the baseline context with the same parameters as the baseline analyses. However, we now clustered the simulated datasets over a range of resolution parameter values (0.1, 0.2, 0.4, 0.8, 1.2, and 2) rather than just the 1.2 presented in the baseline analyses. We performed 100 pre-seeded simulations so that the clustering at each resolution was performed on the same generated datasets. In addition to evaluating power over these different resolutions, we calculated interpretability.

##### Benchmarking analyses

To benchmark scPOST with powsimR and SymSim, we downloaded the powsimR and SymSim packages in R. We split computation times into two parts: parameter estimation and dataset generation (though a whole simulation requires the combination of these times with dataset analysis steps for power estimation). We ran benchmarks in two computational environments: an environment with 16GB RAM and one processor, and a high-performance cluster with 128GB RAM and 24 processors.

For parameter estimation, we downsampled the TB dataset so that it contained 500, 5,000, 25,000, and 50,000 cells. We then estimated parameters on these downsampled datasets using each tool’s corresponding function (*estimateParam* for powsimR, *BestMatchParams* for SymSim, and *estimateFreqVar* and *estimatePCVar* for scPOST) with default parameters. As parameter estimation completed for each tool, we used these parameter estimates as input for each tool’s corresponding dataset simulation functions. We used default parameters for the powsimR and SymSim functions (*Setup* and *simulateDE* for powsimR, and *SimulateTrueCounts* and *True2Observed* for SymSim). For scPOST, we used the *simDataset.base* function with the “clusterData” parameter set to FALSE.

##### Fixed-effects modeling

In order to perform differential abundance with a simple fixed-effects model, we fit a linear regression for each cluster. We predicted the case-control status of a sample by the frequency of that cluster in the sample (predictor) – for each cluster, we received a p value based on significance of the predictor coefficient and performed Bonferroni correction (p < 0.05/*k*) where *k* is the number of clusters. We determined successful detection when at least one cluster was significantly differentially abundant.

##### Estimating power with SC3 clustering

To estimate power to detect DA after using the SC3 clustering algorithm (instead of Louvain) to cluster simulated datasets, we simulated realistic RA datasets with the same parameters as the baseline analyses. After SC3 clustering of the simulated datasets, we performed DA testing as normal. We ran 100 simulations for each combination of parameters.

##### Estimating power with Milo

As an alternative to using MASC for DA testing, we performed a simple demonstration of DA testing with the neighborhood-focused algorithm, Milo. To evaluate power with Milo, we simulated realistic RA datasets with the same parameters as the baseline analyses. To perform Milo DA testing on a simulated dataset, we followed the procedure outlined at: https://rawcdn.githack.com/MarioniLab/miloR/7c7f906b94a73e62e36e095ddb3e3567b414144e/vignettes/milo_gastrulation.html#5_Finding_markers_of_DA_populations. We created a Milo object from the simulated dataset PCs, and ran the following functions with respective parameters: “buildGraph” with k = 10 and d = 20, “makeNhoods” with prop = 0.1, k = 10, d = 20, and refined = TRUE, “countCells” with metadata from the simulated dataset and sample = “sample”, “calcNhoodDistance” with d = 20. To estimate power, we checked each simulation’s Milo results for whether a single neighborhood contained a SpatialFDR value lower than 0.05. We ran 100 simulations for each combination of parameters. For the “cf_scale_ = 0” analysis, we performed the same procedure as just described, but set “cf_scale_ = 0” when simulating datasets.

For determining neighborhood-based inputs, we used Milo to determine neighborhood assignments for each cell. We manually annotated cells to make neighborhoods assignments between cells to be mutually exclusive (each cell is assigned to only one neighborhood). For parameter estimation, we removed cells in neighborhoods that contained <5 cells.

### QUANTIFICATION AND STATISTICAL ANALYSIS

The R package, **scPOST**, was used for generation of single-cell datasets and power analysis. The relevant R packages and specific functions used for each analysis are detailed in the “Method details” section of the STAR Methods. All statistical details for each analysis are detailed in the respective sections in the “Method details” section of the STAR Methods. Values of n represents the number of simulations run (number of n is detailed in figure legends and respective sections in STAR Methods). Dispersion and precision measures are outlined in figure legends. The statistical tests for detecting differential abundance (assessing significance of a simulated dataset) were either MASC or Milo.

## Supplementary Material

1

## Figures and Tables

**Table:** KEY RESOURCES TABLE

REAGENT or RESOURCE	SOURCE	IDENTIFIER
Deposited data		
AMP Rheumatoid Arthritis Phase 1 (processed)	ImmPort	ImmPort: SDY998 (https://www.immport.org/shared/study/SDY998)
AMP Rheumatoid Arthritis Phase 1 (raw)	dbGaP	dbGaP: phs001457.v1.p1 (https://www.ncbi.nlm.nih.gov/projects/gap/cgi-bin/study.cgi?study_id=phs001457.v1.p1)
Human ulcerative colitis single-cell dataset	Single-Cell Portal (Broad Institute)	SCP: SCP259 (https://singlecell.broadinstitute.org/single_cell/study/SCP259/intra-and-inter-cellular-rewiring-of-the-human-colon-during-ulcerative-colitis)
TBRU memory T cell CITE-seq data	GEO	GEO: GSE158769 (https://www.ncbi.nlm.nih.gov/geo/query/acc.cgi?acc=GSE158769)
Software and algorithms		
scPOST (current version)	GitHub	https://github.com/immunogenomics/scpost
scPOST (repository)	Zenodo	https://doi.org/10.5281/zenodo.5573126

## Highlights

- single-cell POwer Simulation Tool (scPOST) is a fast and flexible simulation framework
- scPOST allows users to compare study designs to assess how they affect detection power
- Increasing sample size improves power more than increasing cells per sample

## References

[R1] Blondel VD, Guillaume JL, Lambiotte R, and Lefebvre E (2008). Fast unfolding of communities in large networks. J. Stat. Mech 10, P10008.

[R2] Chen HK, Boettiger AN, Moffitt JR, Wang S, and Zhuang X (2015). Spatially resolved, highly multiplexed RNA profiling in single cells. Science 348, aaa6090. 25858977 10.1126/science.aaa6090 PMC4662681

[R3] Cusanovich DA, Daza R, Adey A, Pliner H, Christiansen L, Gunderson KL, Steemers FJ, Trapnell C, and Shendure J (2015). Multiplex single cell profiling of chromatin accessibility by combinatorial cellular indexing. Science 348, 910–914. 25953818 10.1126/science.aab1601 PMC4836442

[R4] Dann E, Henderson NC, Teichmann SA, Morgan MD, and Marioni JC (2020). *Milo*: differential abundance testing on single-cell data using k-NN graphs. bioRxiv. 10.1101/2020.11.23.393769. PMC7617075 34594043

[R5] Van Der Maaten L, and Hinton G (2008). Visualizing high-dimensional data using t-SNE. J. Mach Learn Res 9, 2579–2605.

[R6] Fonseka CY, Rao DA, Teslovich NC, Korsunsky I, Hannes S, Slowikowski K, Gurish MF, Donlin LT, Lederer JA, Weinblatt ME, (2018). Mixed-effects association of single cells identifies an expanded effector CD4+ T cell subset in rheumatoid arthritis. Sci. Transl Med 10, eaaq0305. 30333237 10.1126/scitranslmed.aaq0305 PMC6448773

[R7] Gayoso A, Lopez R, Steier Z, Regier J, Streets A, and Yosef N (2019).A joint model of RNA expression and surface protein abundance in single cells. bioRxiv. 10.1101/791947.

[R8] Gayoso A, Steier Z, Lopez R, Regier J, Nazor K, Streets A, and Yosef N (2020). Joint probabilistic modeling of paired transcriptome and proteome measurements in single cells. bioRxiv. 10.1101/2020.05.08.083337. PMC7954949 33589839

[R9] Haghverdi L, Buttner M, Wolf FA, Buettner F, and Theis FJ (2016). Diffusion pseudotime robustly reconstructs lineage branching. Nat. Methods 13, 845–848. 27571553 10.1038/nmeth.3971

[R10] Haghverdi L, Lun ATL, Morgan MD, and Marioni JC (2018). Batch effects in single-cell RNA-sequencing data are corrected by matching mutual nearest neighbors. Nat. Biotechnol 36, 421–427. 29608177 10.1038/nbt.4091 PMC6152897

[R11] Hollenbach JA, Mack SJ, Thomson G, and Gourraud PA (2014). Analytical methods for disease association studies with immunogenetic data. Methods Mol. Biol 882, 245–266. 10.1007/978-1-61779-842-9_14 PMC4209949 22665238

[R12] Jaitin DA, Kenigsberg E, Keren-Shaul H, Elefant N, Paul F, Zaretsky I, Mildner A, Cohen N, Jung S, Tanay A, (2014). Massively parallel single-cell RNA-seq for marker-free decomposition of tissues into cell types. Science 343, 776–779. 24531970 10.1126/science.1247651 PMC4412462

[R13] Kiselev VY, Kirschner K, Schaub MT, Andrews T, Yiu A, Chandra T, Natarajan KN, Reik W, Barahona M, Green AR, (2017). SC3: consensus clustering of single-cell RNA-seq data. Nat. Methods 14, 483–486. 28346451 10.1038/nmeth.4236 PMC5410170

[R14] Korsunsky I, Millard N, Fan J, Slowikowski K, Zhang F, Wei K, Baglaenko Y, Brenner M, Loh P-R, and Raychoudhuri S (2019). Fast, sensitive and accurate integration of single-cell data with Harmony. Nat. Methods 16, 1289–1296. 31740819 10.1038/s41592-019-0619-0 PMC6884693

[R15] Kotliarov Y, Sparks R, Martins AJ, Mulè MP, Lu Y, Goswami M, Kardava L, Banchereau R, Pascual V, Biancoto A, (2020). Broad immune activation underlies shared set point signatures for vaccine responsiveness in healthy individuals and disease activity in patients with lupus. Nat. Med 26, 618–629. 32094927 10.1038/s41591-020-0769-8 PMC8392163

[R16] Levine JH, Simonds EF, Bendall S, Davis KL, Amir ED, Tadmor MD, Litvin O, Feinberg HG, Jager A, Zunder ER, (2015). Data-driven phenotypic dissection of aml reveals progenitor-like cells that correlate with prognosis. Cell 162, 184–197. 26095251 10.1016/j.cell.2015.05.047 PMC4508757

[R17] Li WV, and Li JJ (2019).A statistical simulator scDesign for rational scRNA-seq experimental design. Bioinformatics 35, i41–i50. 31510652 10.1093/bioinformatics/btz321 PMC6612870

[R18] Luecken MD, and Theis FJ (2019). Current best practices in single-cell RNA-seq analysis: a tutorial. Mol. Syst. Biol 15, e8746. 31217225 10.15252/msb.20188746 PMC6582955

[R19] Mandric I, Schwarz T, Majumdar A, Kangcheng H, Briscoe L, Perez R, Subramaniam M, Hafemeister C, Satija R, Ye CJ, (2020). Optimized design of single-cell RNA sequencing experiments for cell-type-specific eQTL analysis. Nat. Commun 11, 5504. 33127880 10.1038/s41467-020-19365-w PMC7599215

[R20] McInnes L, Healy J, and Melville J (2018). UMAP: Uniform Manifold Approximation and Projection for Dimension Reduction. https://arxiv.org/abs/1802.03426.

[R21] Nathan A, Beynor JI, Baglaenko Y, Suliman S, Ishigaki K, Asgari S, Huang C-C, Luo Y, Shang Z, Lopez K, (2021). Multimodally profiling memory T cells from a tuberculosis cohort identifies cell state associations with demographics, environment, and disease. Nat. Immunol 22, 781–793. 34031617 10.1038/s41590-021-00933-1 PMC8162307

[R22] Orrù V, Steri M, Sole G, Sidore C, Virdis F, (2013). Genetic variants regulating immune cell levels in health and disease. Cell 155, 242–256. 24074872 10.1016/j.cell.2013.08.041 PMC5541764

[R23] Pollen A, Nowakowski T, Shuga J, Wang X, Leyrat AA, Lui JH, Li N, Szpankowski L, Fowler B, Chen P, (2014). Low-coverage single-cell mRNA sequencing reveals cellular heterogeneity and activated signaling pathways in developing cerebral cortex. Nat. Biotechnol 32, 1053–1058. 25086649 10.1038/nbt.2967 PMC4191988

[R24] Qiu X, Mao Q, Tang Y, Wang L, Chawla R, Pliner HA, and Trapnell C (2017). Reversed graph embedding resolves complex single-cell trajectories. Nat. Methods 14, 979–982. 28825705 10.1038/nmeth.4402 PMC5764547

[R25] Regev A, Teichmann SA, Lander ES, Amit I, Benoist C, Birney E, Bodenmiller B, Campbell P, Carninci P, Clatworthy M, (2017). Science forum: the human cell atlas. eLife 6, e27041. 29206104 10.7554/eLife.27041 PMC5762154

[R26] Reyes M, Filbin MR, Bhattacharyya RP, Billman K, Eisenhaure T, Hung DT, Levy BD, Baron RM, Blainey PC, Goldberg MB, (2020). An immune-cell signature of bacterial sepsis. Nat. Med 26, 333–340. 32066974 10.1038/s41591-020-0752-4 PMC7235950

[R27] Rodriques SG, Stickels RR, Goeva A, Martin CA, Murray E, Vanderburg CR, Welch J, Chen LM, Chen F, and Macosko EZ (2019). Slide-seq: a scalable technology for measuring genome-wide expression at high spatial resolution. Science 363, 1463–1467. 30923225 10.1126/science.aaw1219 PMC6927209

[R28] Schafflick D, Xu CA, Hartlehnert M, Cole M, Schulte-Mecklenbeck A, Lautwein T, Wolbert J, Heming M, Meuth SG, Kuhlmann T, (2020). Integrated single cell analysis of blood and cerebrospinal fluid leukocytes in multiple sclerosis. Nat. Commun 11, 247. 31937773 10.1038/s41467-019-14118-w PMC6959356

[R29] Schmid KT, Cruceanu C, Böttcher A, Lickert H, Binder EB, Theis FJ, and Heinig M (2020). Design and power analysis for multi-sample single cell genomics experiments. Single Cell Genomics. 10.1101/2020.04.01.019851. PMC8595682 34785648

[R30] Shalek AK, Satija R, Shuga J, Trombetta JJ, Gennert D, Raychowdhury R, Schwartz S, Yosef N, Malboeuf C, Lu D, (2014). Single-cell transcriptomics reveals bimodality in expression and splicing in immune cells. Nature 498, 236–240. 10.1038/nature12172 PMC3683364 23685454

[R31] Smillie CS, Biton M, Ordovas-Montanes J, Sullivan KM, Burgin G, Graham DB, Herbst RH, Rogel N, Slyper M, Waldman J, (2019). Intra- and inter-cellular rewriting of the human colon during ulcerative colitis. Cell 178, 714–730. 31348891 10.1016/j.cell.2019.06.029 PMC6662628

[R32] Soneson C, and Robinson MD (2018). Bias, robustness and scalability in single-cell differential expression analysis. Nat. Methods 15, 255–261. 29481549 10.1038/nmeth.4612

[R33] Stoeckius M, Hafemeister C, Stephenson W, Houck-Loomis B, Chattopadhyay PK, Swerdlow H, Satija R, and Smibert P (2017). Simultaneous epitope and transcriptome measurement in single cells. Nat. Methods 14, 865–868. 28759029 10.1038/nmeth.4380 PMC5669064

[R34] Stuart T, and Satija R (2019). Integrative single-cell analysis. Nat. Rev. Genet 20, 257–272. 30696980 10.1038/s41576-019-0093-7

[R35] Svensson V, Vento-Tormo R, and Teichmann SA (2018). Exponential scaling of single-cell RNA-seq in the past decade. Nat. Protoc 13, 599–604. 29494575 10.1038/nprot.2017.149

[R36] Tanay A, and Regev A (2017). Single cell genomics: from phenomenology to mechanism. Nature 541, 331–338. 28102262 10.1038/nature21350 PMC5438464

[R37] Tang F, Barbacioru C, Wang Y, Nordman E, Lee C, Xu N, Wang X, Bodeau J, Touch BB, Siddiqui A, (2009). mRNA-Seq whole-transcriptome analysis of a single cell. Nat. Methods 6, 377–382. 19349980 10.1038/nmeth.1315

[R38] Vieth B, Zieganhain C, Parekh S, Enard W, and Hellmann I (2017). pows-imR: power analysis for bulk and single cell RNA-seq experiments. Bioinformatics 33, 3486–3488. 29036287 10.1093/bioinformatics/btx435

[R39] Zappia L, Phipson B, and Oshlack A (2017). Splatter: simulation of single-cell RNA sequencing data. Genome Biol. 18, 174. 28899397 10.1186/s13059-017-1305-0 PMC5596896

[R40] Zhang F, Wei K, Slowikowski K, Fonseka C, Rao DA, Kelly S, Goodman SM, Tabechian DE, Hughes LB, Saloman-Escoto K, (2019a). Defining inflammatory cell states in rheumatoid arthritis joint synovial tissues by integrating single-cell transcriptomics and mass cytometry. Nat. Immunol. 20, 928–942. 31061532 10.1038/s41590-019-0378-1 PMC6602051

[R41] Zhang X, Xu C, and Yosef N (2019b). Simulating multiple faceted variability in single cell RNA sequencing. Nat. Commun 10, 2611. 31197158 10.1038/s41467-019-10500-w PMC6565723

