# Supplementary material for: Maximizing statistical power to detect differentially abundant cell states with scPOST
Source: Cell Rep Methods. 2021 Nov 22;1(8):100120. doi: 10.1016/j.crmeth.2021.100120 (PMC8740883; doi:10.1016/j.crmeth.2021.100120)
Supplement: Document S1. Figures S1–S9 and Tables S1 and S2 [file mmc1.pdf]

Cell Reports Methods, Volume *1*

Supplemental information

Maximizing statistical power to detect differentially abundant cell states with scPOST

Nghia Millard, Ilya Korsunsky, Kathryn Weinand, Chamith Y. Fonseka, Aparna Nathan, Joyce B. Kang, and Soumya Raychaudhuri

Supplementary Figures and Tables

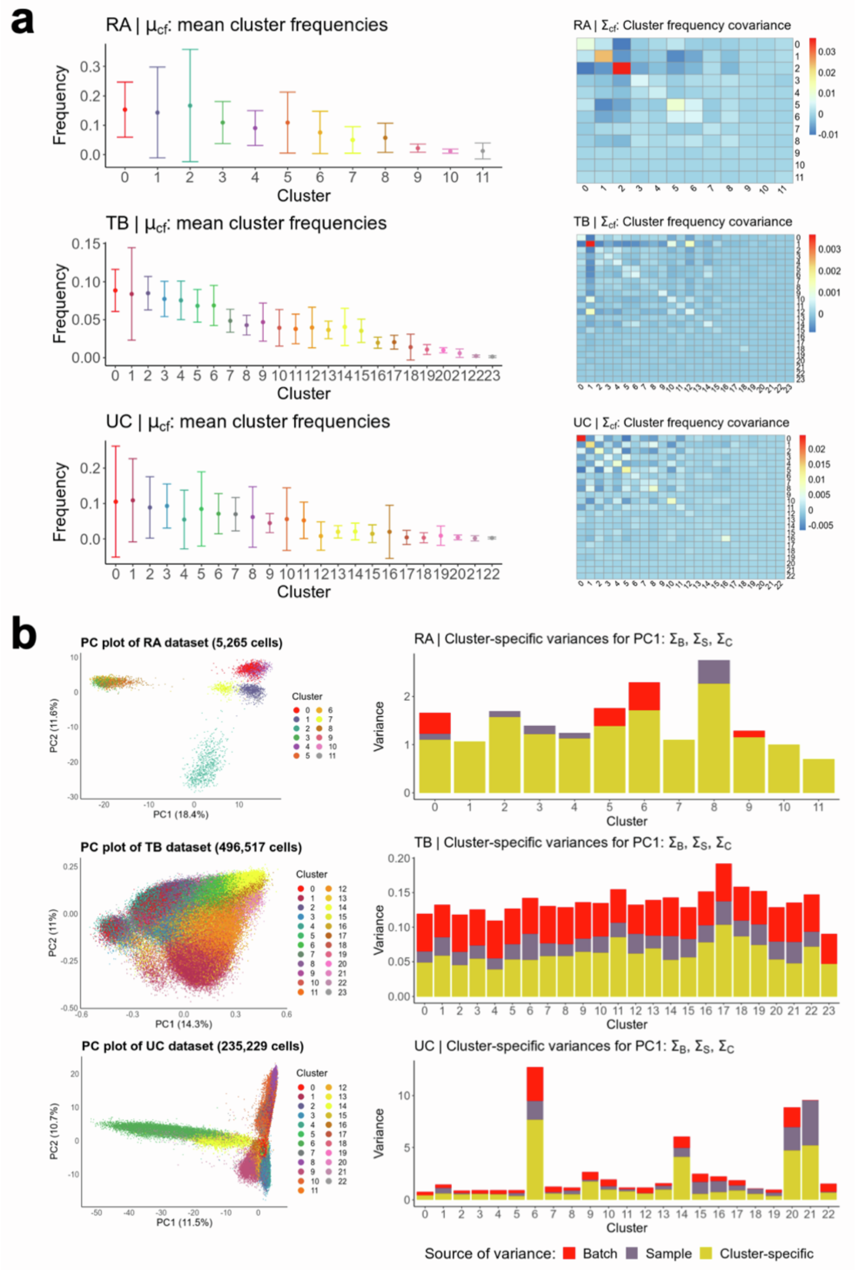

Supplemental Figure 1 | Cluster frequency variation across samples and principal component structure of the RA, TB, and UC datasets highlight experimental variability, related to Figure 3. Panel a, left: Frequency distribution for each cluster in the RA, TB, and UC datasets. Plotted mean frequencies are the observed mean frequency of that cluster across all samples in their respective dataset. In all panels, error bars represent one standard deviation from the mean in each direction to showcase the spread of frequencies across samples. **Panel a, right:** Cluster frequency covariance matrices for the RA, TB, and UC datasets. **Panel b, left,** PC plots of the RA, TB, and UC datasets colored by cluster. **Panel b, right,** Bar plots of the estimated variance in each cluster for the first principal component in each of the RA, TB, and UC datasets. Each bar is colored by the estimated proportion of variance that each source contributes, as estimated by scPOST.

	RA dataset	TB dataset	UC dataset
Single-cell technology	CelSeq2[41]	10X Chromium 3’ v3	10X Chromium 3’ v2/v3
Number of samples	21	259	30
Number of cells passed QC	5,265	496,517	235,229
Type of samples	Synovial tissue (joint replacement procedure or biopsy)	PBMCs (blood)	Intestinal biopsy
Broad cell types assayed	Immune and stromal cells (T/B cells, Monocytes, Fibroblasts)	Memory T cells	Immune and stromal cells (T/B/Myeloid cells, Fibroblasts, Endothelial cells, Epithelial cells)
Number of clusters	12	24	23
Mean UMI/cell	7,300	4,920	4,582
Mean unique genes/cell	2,432	1,472	988
Batch-correction	Harmony	None	None

Supplemental Table 1 | Characteristics of the RA, TB, and UC datasets, related to Figure 3.

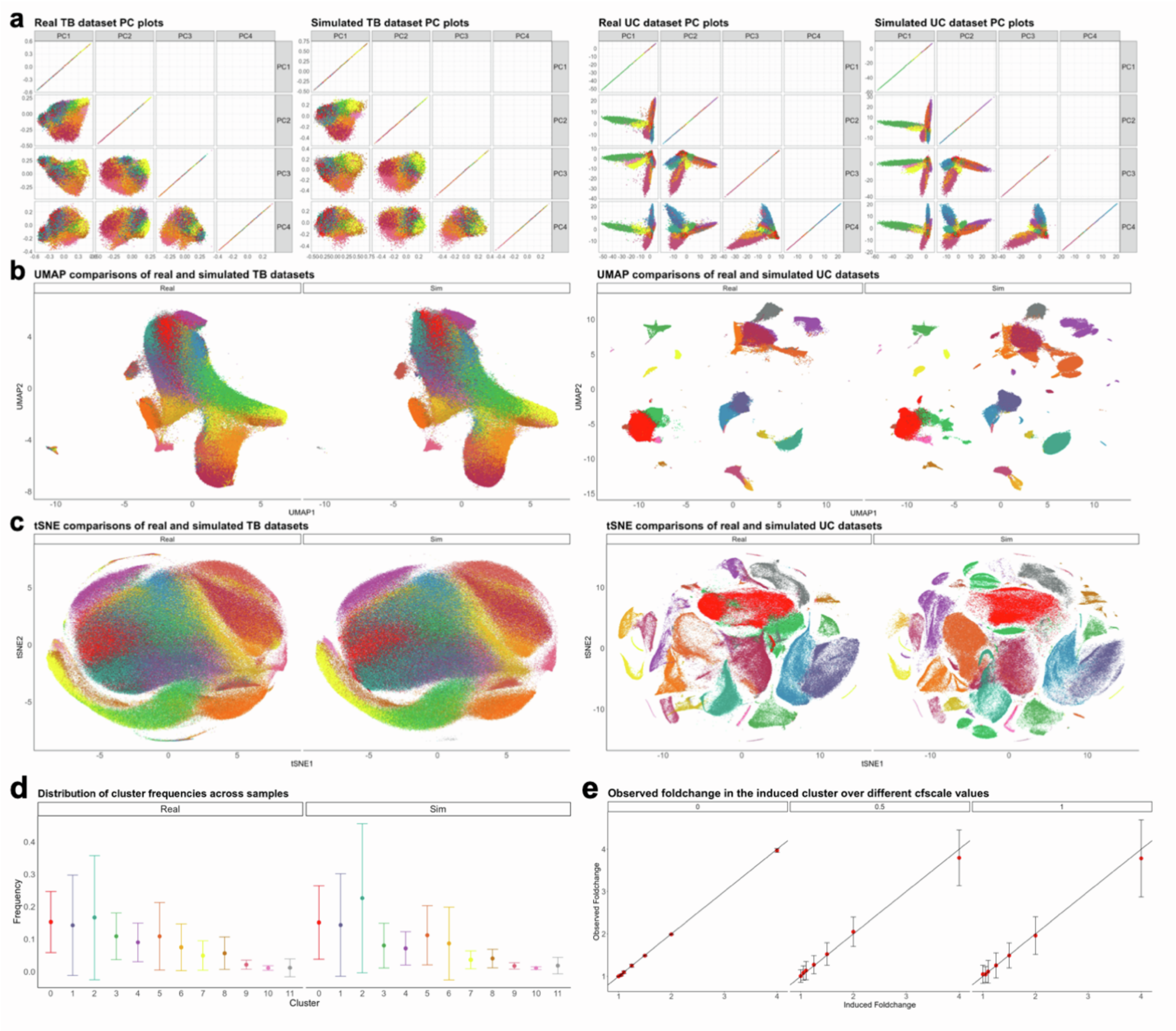

Supplemental Figure 2 | scPOST can generate multi-sample single-cell datasets with gene expression and cluster frequency distributions similar to the input dataset, related to Figure 4. a-c, Each panel contains visualizations comparing the PC, UMAP, or tSNE structure between a simulated TB/UC dataset and the respective real dataset. UMAP and tSNE embeddings were created from embedding the PCs of the real and simulated dataset into the same 2-dimensional space. **d,** Frequency distribution for each cluster in the real RA dataset and an example simulated realistic dataset. Error bars represent one standard deviation from the mean to showcase the spread of frequencies across samples. **e,** Dot-plots comparing the intended induced fold changes with the actual observed fold changes in simulations. Each point represents the mean observed fold change over 100 simulations. Error bars represent one standard deviation from the mean to illustrate the spread of observed fold changes, and how that spread decreases as cfscale values decrease.

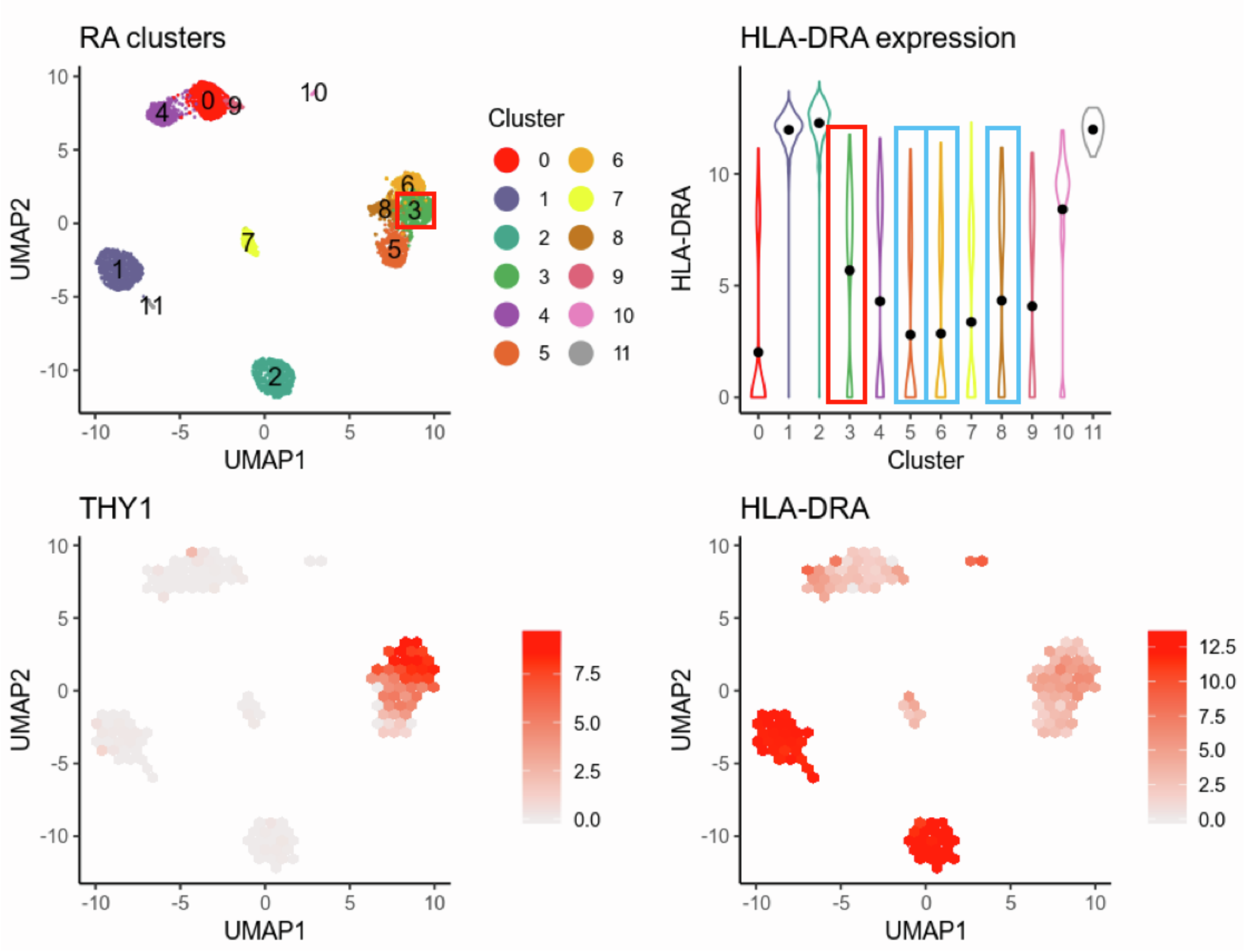

Supplemental Figure 3 | The RA dataset contains THY1+ sub-lining fibroblasts that express HLA-DRA, related to Figure 5. For the analyses in which we expanded the RA study, we focused on the THY1+ sub-lining fibroblast cluster (cluster 3 highlighted in red, other fibroblast clusters highlighted in blue) that most highly expressed *HLA-DRA*, which corresponds to the HLA-DRA^{hi} fibroblast population described in the paper. The RA dataset was filtered to only include fibroblasts before inputting into scPOST for **Fig. 5a**.

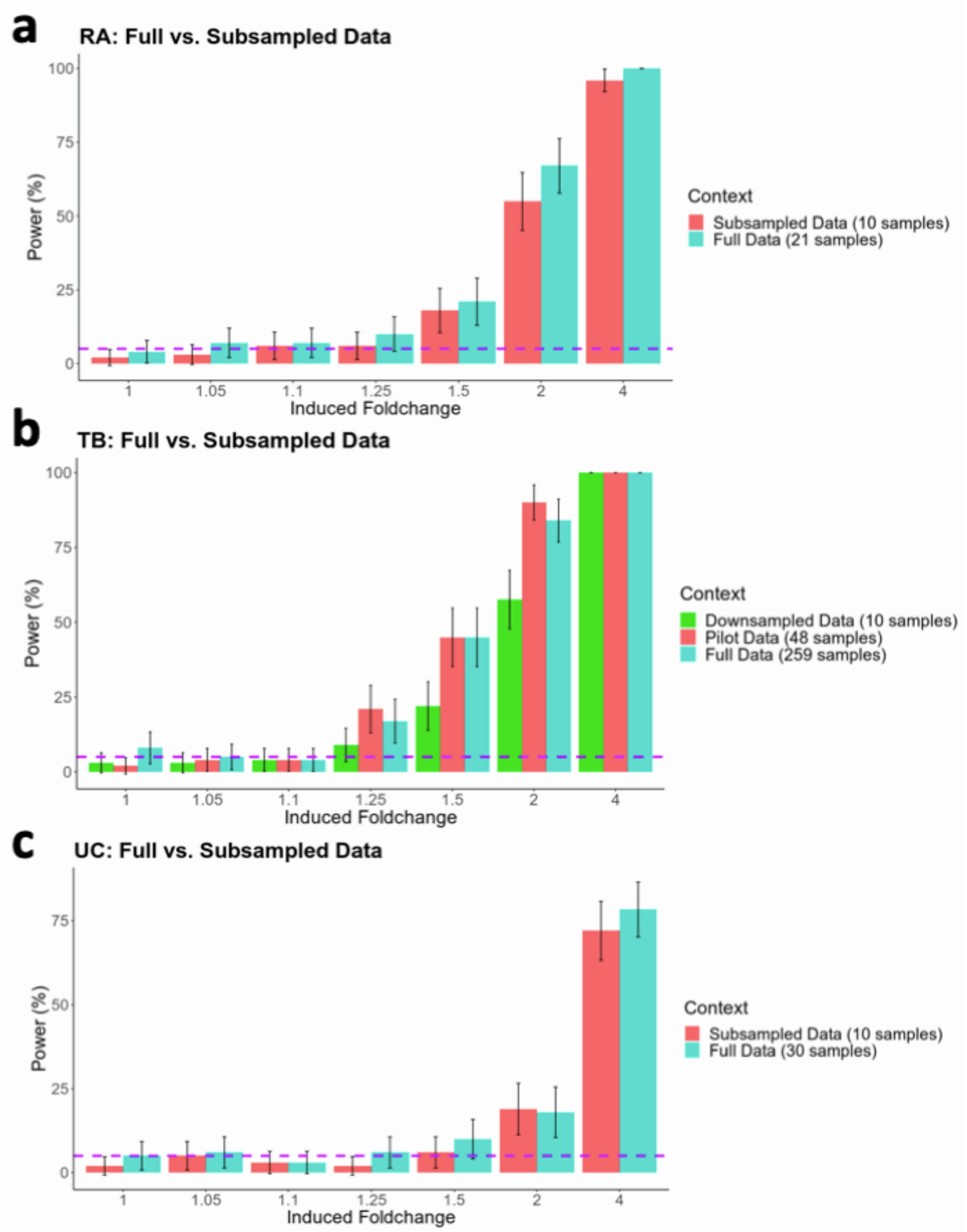

Supplemental Figure 4 | Larger input dataset sizes tend to provide more accurate parameter estimates, leading to increased power, related to Figure 5. a-c, We sub-sampled each independent dataset by randomly sampling a specific number of samples, and then input these sub-sampled datasets into scPOST. Comparing the estimated power of smaller-sized data with the power retrieved from the full data suggests that increasing the size of the input can lead to more accurate power estimates.

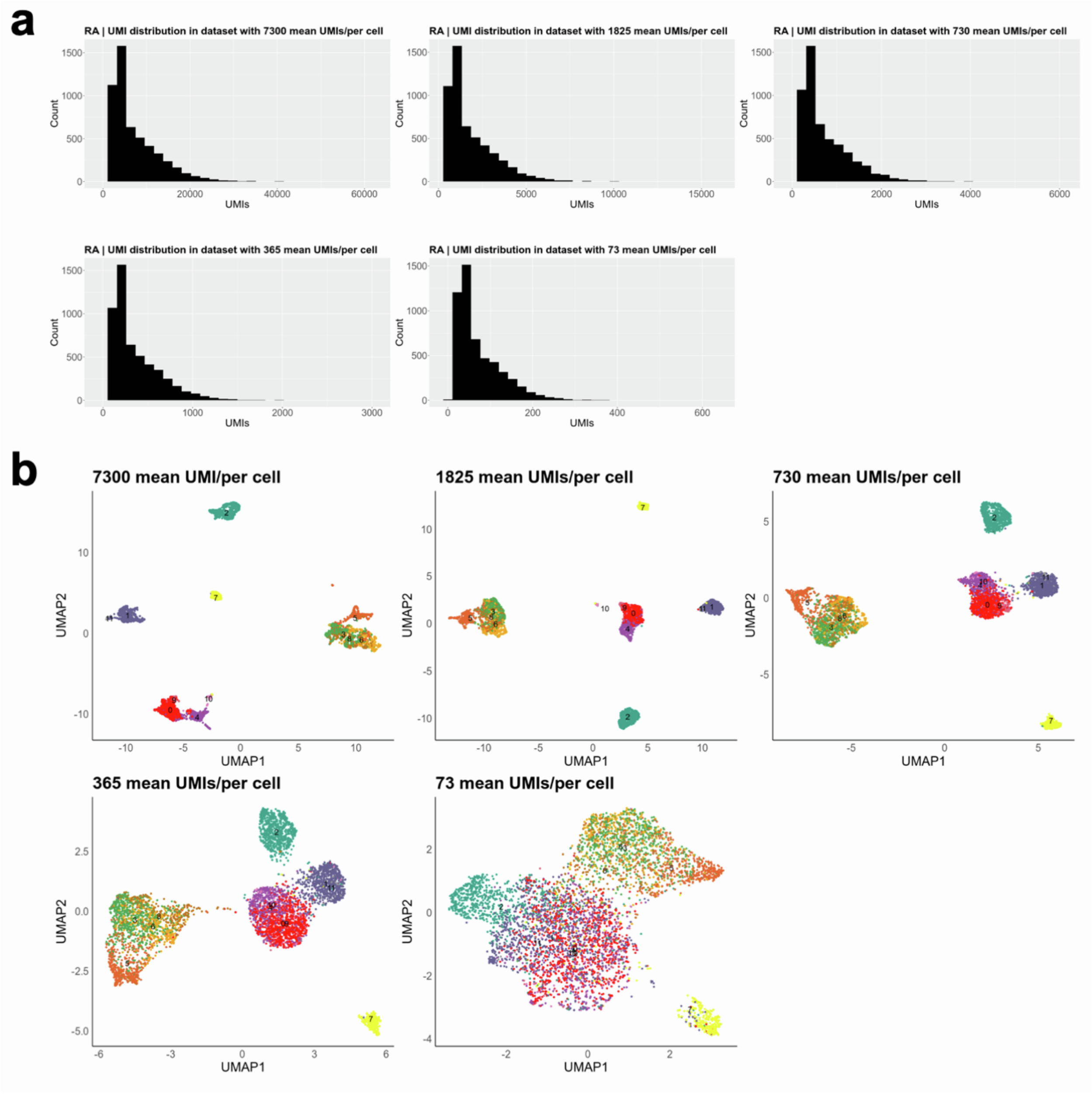

Supplemental Figure 5 | The real RA dataset (7300 mean UMIs/per cell) was binomially downsampled to create input datasets with varying levels of mean UMIs, related to Figure 5. a, Histogram of the UMI distribution of the original RA dataset and the datasets whose UMIs were downsampled. Downsampled datasets maintained similar sequencing read distributions, but featured a lower mean number of reads. **b**, Downsampled datasets were input into standard PCA dimensionality reduction pipeline, and then visualized with UMAP. Cells are colored by the cell state identities obtained from the original RA dataset.

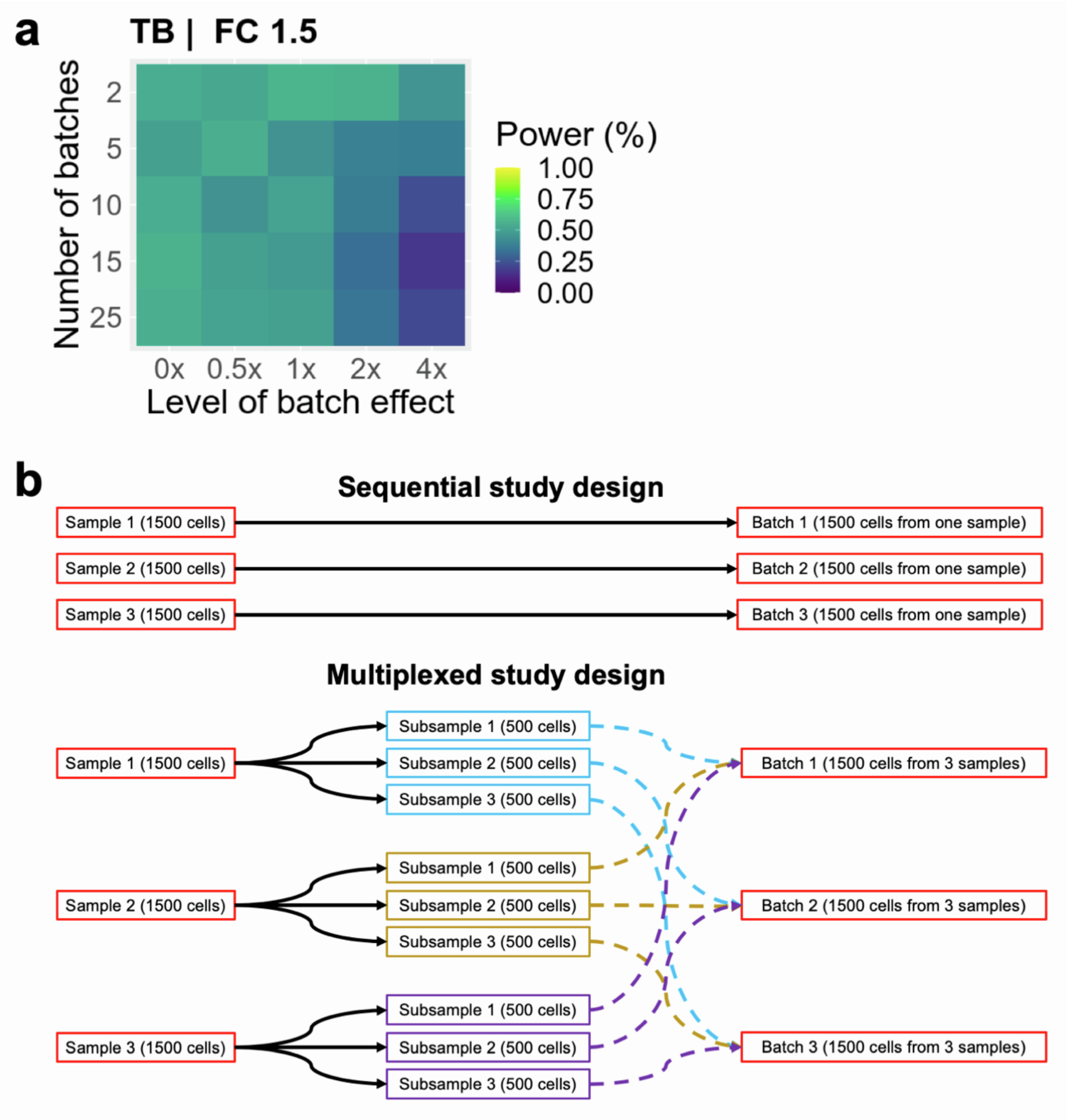

Supplemental Figure 6 | scPOST facilitates exploration of different study designs, such as batch multiplexing structures, related to Figure 6. a, Power calculations across different ranges of scaled batch effects and number of batches, with the induced fold change set to 1.5. Simulations were performed in the realistic context, but with modulated levels of batch-influenced variation on gene expression. The number of batches decreasing indicates increasing the number of samples run in a batch. **b,** In **Fig. 6a**, we compare the estimated power from two batch multiplexing structures with scPOST. The non-multiplexed sequential design placed each simulated sample in its own batch, so that each batch contained cells from only one sample. For the sequential design in **Fig. 6a**, we placed samples (total of 100 with 2000 cells each) into 100 batches. The multiplexed study design featured a batch structure in which each simulated sample was split into equally-sized subsamples. These subsamples were then placed into different batches, so that each batch contained cells from multiple samples. For the multiplexed design in **Fig. 6a**, we split each sample (total of 100 with 2000 cells each) into 4 subsamples (500 cells each), which were then placed into 100 batches (each batch contained cells from different subsamples).

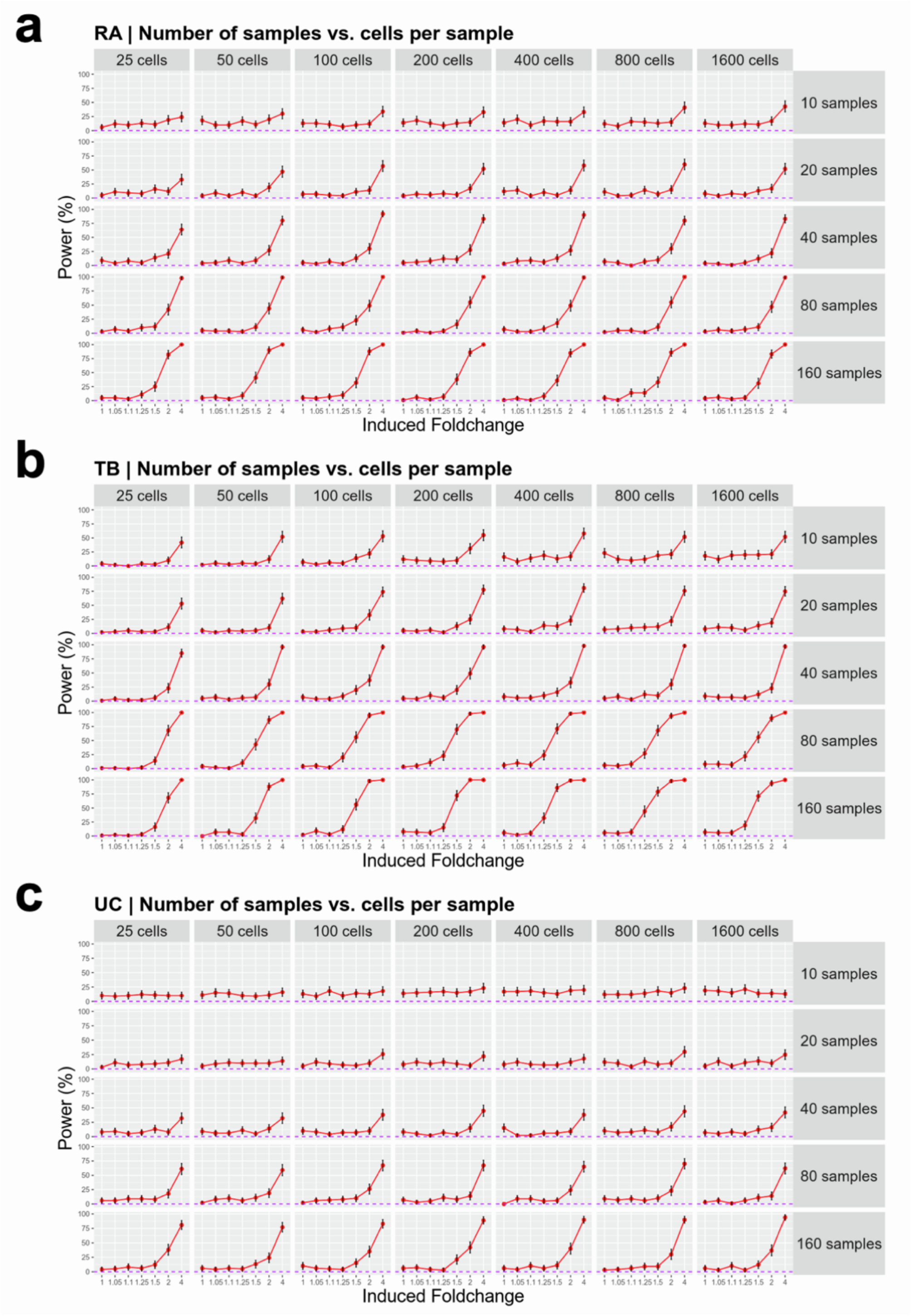

Supplemental Figure 7 | Power estimations in the RA, TB, and UC settings for varying dataset sizes characterized by a variable number of samples and cells per sample, related to Figure 6. Power estimations for different sample/cell per sample combinations. Simulations were performed in the baseline context (**Fig. 5b**) with each data point representing 100 simulations. Grid elements along a top right-bottom left diagonal represent an equivalent number of cells. Error bars represent 95% binomial proportion confidence intervals and the dotted horizontal purple line represents 5% power.

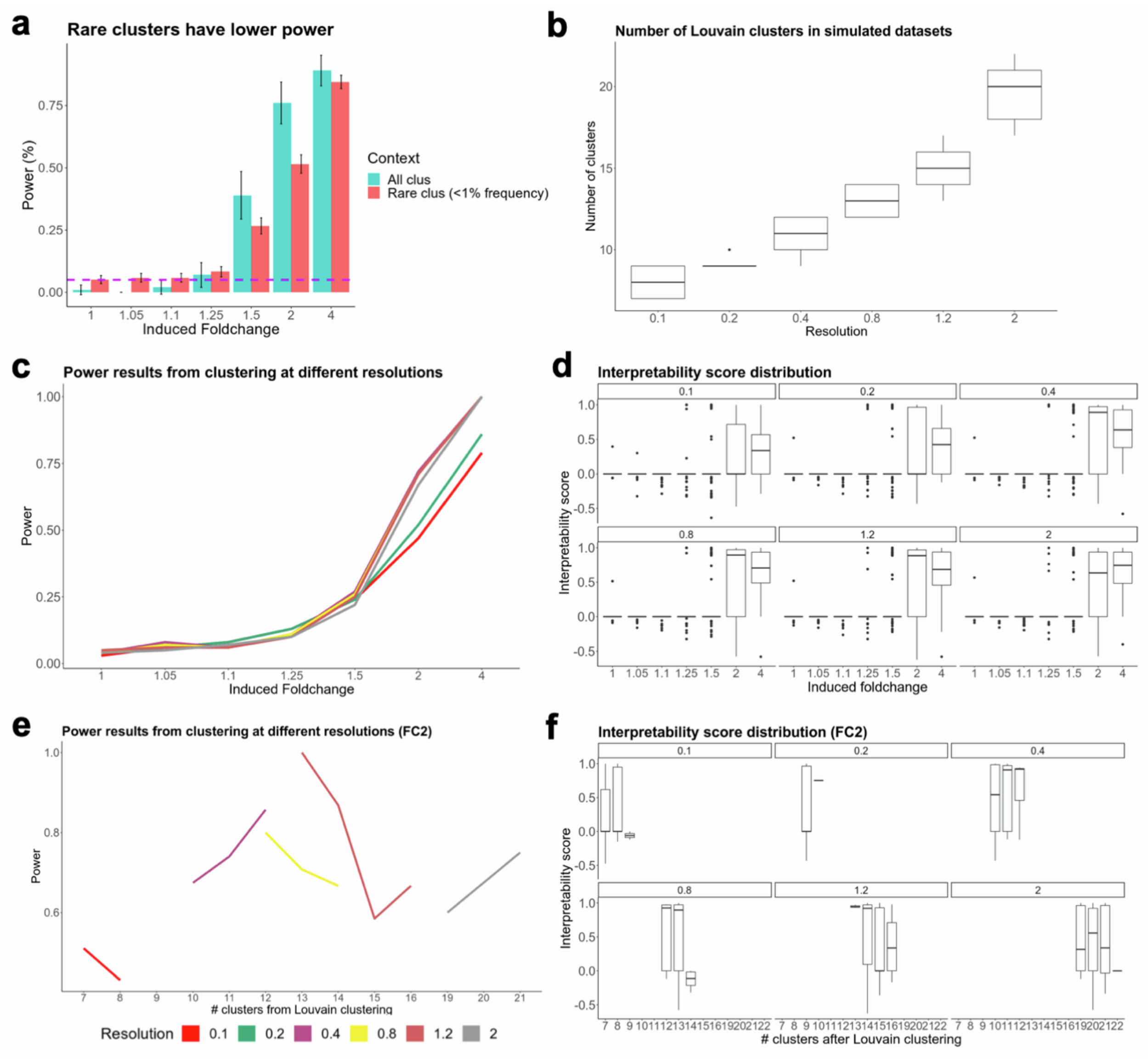

Supplemental Figure 8 | Power estimates decreases in lower frequency clusters (<1% mean frequency across samples), and vary across a range of Louvain clustering resolution values, related to STAR Methods. a, Power estimates performed in the realistic TB context. Comparison between detecting effects across the frequency spectrum (All clus), and focusing on detecting effects that were only induced in rare clusters (Rare clus). **b,** Boxplots showing the spread of the number of clusters obtained from Louvain clustering simulated data. Each boxplot represents results from 100 pre-seeded simulations. **c,** Line plots showing power results from clustering 100 pre-seeded simulations at different resolutions. **d,** Zoom-in on FC2 results (100 simulations) as shown in **Fig. S8c**. Results are specific to number of clusters obtained from Louvain clustering in the simulation, as well as the resolution parameter value used for the Louvain clustering. **e,** Boxplots showing the spread of interpretability scores for 100 pre-seeded simulations each. **f,** Zoom-in on FC2 results (100 simulations) as shown in **Fig. S8e**. Boxplots are specific to number of clusters obtained from Louvain clustering in the simulation, as well as the resolution parameter value used for the Louvain clustering.

Context: Full dataset (30K genes)

Computation time (seconds) for parameter estimation (16GB)

	scPOST	powsimR	symSim
500 cells	26s	20s	28s
5000 cells	27s	27s	35s
25,000 cells	27s	442s	40s
50,000 cells	34s	OoM	68s

Computation time (seconds) for parameter estimation (128GB)

	scPOST	powsimR	symSim
500 cells	25s	28s	28s
5000 cells	27s	365s	34s
25,000 cells	27s	384s	35s
50,000 cells	33s	883s	39s

Computation time (seconds) for dataset generation (16GB)

	scPOST	powsimR	symSim
500 cells	1s	28s	6033s
5000 cells	2s	748s	OoM
25,000 cells	8s	OoM	OoM
50,000 cells	19s	OoM	OoM

Computation time (seconds) for dataset generation (128GB)

	scPOST	powsimR	symSim
500 cells	1s	28s	4073s
5000 cells	2s	713s	58,876s
25,000 cells	8s	OoM	OoM
50,000 cells	15s	OoM	OoM

Context: Fewer genes (2K genes)

Computation time (seconds) for dataset generation (16GB)

	scPOST	powsimR	symSim
500 cells	1s	14s	54s
5000 cells	2s	285s	1834s
25,000 cells	8s	589s	21,387s
50,000 cells	19s	OoM	21,427s

Computation time (seconds) for dataset generation (128GB)

	scPOST	powsimR	symSim
500 cells	1s	12s	207s
5000 cells	2s	275s	1,834s
25,000 cells	8s	581s	21,779s
50,000 cells	15s	1006s	22,117s

Supplemental Table 2 | Computational runtimes for running the parameter estimation and dataset generation steps of three single-cell dataset simulation tools: scPOST, powsimR, and symSim, related to STAR methods. Standard parameter estimation and dataset generation functions for each tool were run with default parameters for datasets of the following sizes: 500, 5000, 25,000, and 50,000 cells. We ran simulations on two computational environments: a 16GB RAM and 1 processor machine and a 128GB RAM and 24 processor cluster. OoM = Out of memory, meaning that the simulation failed to complete due to memory constraints.

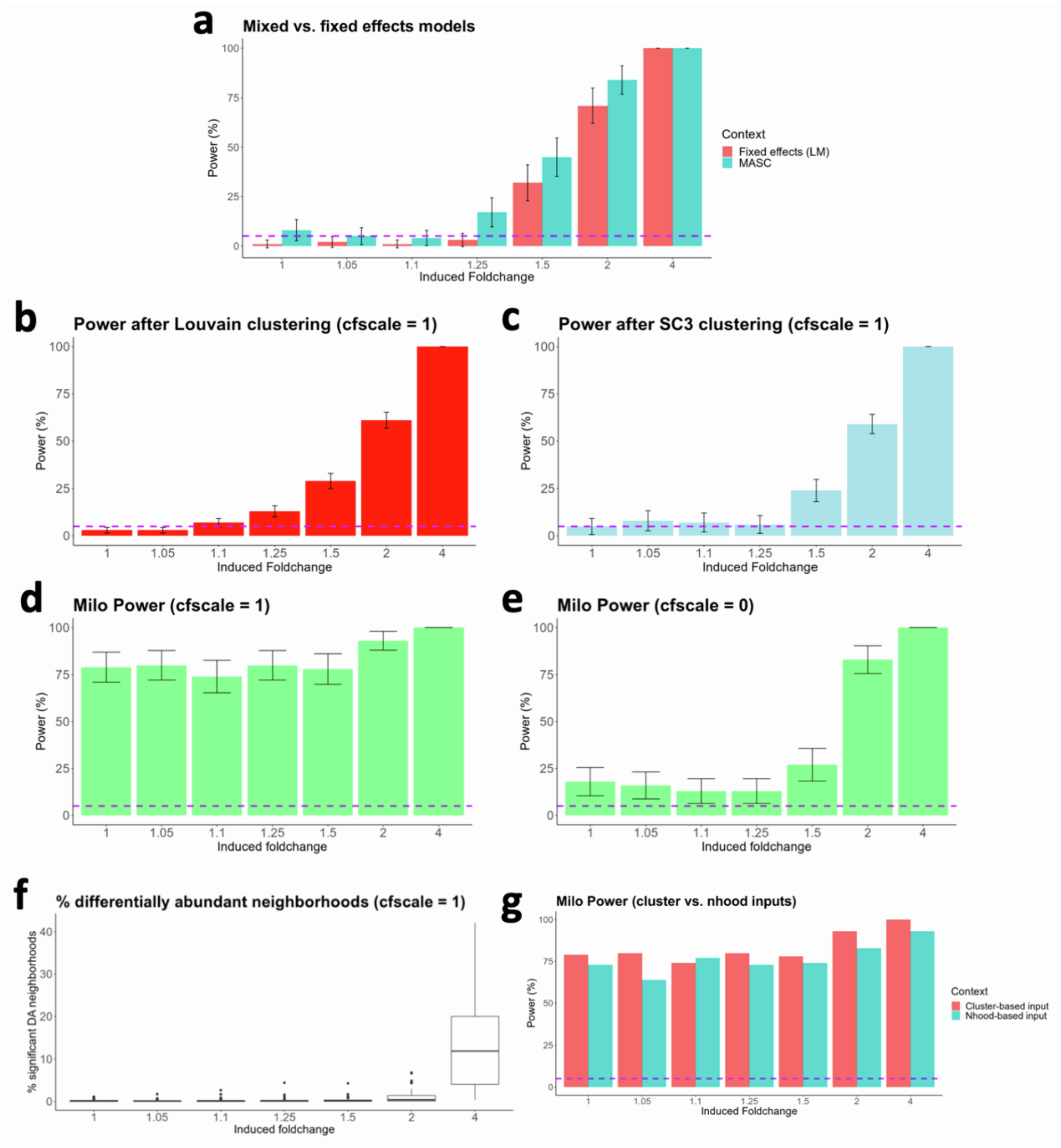

Supplemental Figure 9 | Simulated datasets generated by scPOST are compatible with alternative models, especially those that utilize principal components as input, related to STAR methods. a, Instead of utilizing MASC, we tested for differential abundance with simple linear models that associated the abundance of a cluster in a sample with the case-control status. **b-e**, Error bars represent 95% binomial proportion confidence intervals and the dotted horizontal purple line is set at 5%. Each bar represents results from (n = 100) simulations. **b,** Power results from simulations in the baseline context. Simulated datasets were clustered with Louvain clustering algorithm. **c,** Power from the same simulations in panel **b**, but datasets were clustered with the SC3 clustering algorithm. **d,** Power results from simulations in the baseline context, but using Milo for differential abundance (DA) testing instead of MASC**. e,** Power results in the same context as c, but with cfscale = 0 (no cluster frequency variation between samples)**. f,** Boxplots showing the spread of the percent of DA neighborhoods over many simulations (cfscale = 1). **g,** For the RA dataset, we utilized Milo to determine mutually-exclusive neighborhood membership for each cell (instead of a cluster membership), which we then input into scPOST to estimate power.
